# Post‐COVID Fatigue Is Associated With Reduced Cortical Thickness After Hospitalization

**DOI:** 10.1002/acn3.70260

**Published:** 2025-11-25

**Authors:** Tim J. Hartung, Florentin Steigerwald, Amy Romanello, Cathrin Kodde, Matthias Endres, Sandra Frank, Peter Heuschmann, Philipp Koehler, Stephan Krohn, Daniel Pape, Jens Schaller, Sophia Stöcklein, Istvan Vadasz, Janne Vehreschild, Martin Witzenrath, Thomas Zoller, Carsten Finke, the NAPKON Study Group, Y. Ahlgrimm, C. Finke, J. Fricke, T. Keil, L. Krist, N. Lisewsky, M. Mittermaier, M. Mueller‐Plathe, C. Pley, S. Schmidt, A. Stege, F. Steinbeis, S. Steinbrecher, C. Wildberg, M. Witzenrath, E. Zessin, T. Zoller, C. Arendt, C. Bellinghausen, S. Cremer, A. Groh, A. Gruenewaldt, Y. Khodamoradi, S. Klinsing, G. Rohde, M. Vehreschild, T. Vogl, S. Frank, J. C. Hellmuth, M. Huber, S. Kaeaeb, O. T. Keppler, E. Khatamzas, C. Mandel, S. Mueller, M. Muenchhoff, L. Reeh, C. Scherer, H. Stubbe, M. von Bergwelt, L. Weiss, B. Zwissler, S. Cleef, M. E. Figuera Basso, J. Franzenburg, K. Franzpoetter, A. Friedrichs, A. Hermes, J. Heyckendorf, C. Kujat, I. Lehmann, C. Maetzler, S. Meier, D. Pape, S. Poick, L. Reinke, A. K. Russ, A. M. Scheer, D. Schunk, T. Tamminga, S. Bohnet, D. Droemann, K. F. Franzen, R. Hoerster, N. Kaeding, M. Nissen, P. Parschke, J. Rupp, S. Caesar, H. Einsele, S. Frantz, A. Frey, A. Grau, K. Haas, C. Haertel, K. G. Haeusler, G. Hein, J. Herrmann, A. Horn, R. Jahns, P. Meybohm, F. A. Montellano, C. Morbach, J. Schmidt, P. Schulze, S. Stoerk, J. Volkmann, T. Bahmer, A. Hermes, M. Krawczak, W. Lieb, S. Schreiber, T. Tamminga, B. Balzuweit, S. Berger, J. Fricke, M. Hummel, A. Krannich, L. Krist, F. Kurth, J. Lienau, R. Lorbeer, C. Pley, J. Schaller, S. Schmidt, C. Thibeault, M. Witzenrath, T. Zoller, I. Bernemann, T. Illig, M. Kersting, N. Klopp, V. Kopfnagel, S. Muecke, M. Kraus, B. Lorenz‐Depiereux, G. Anton, A. Kuehn‐Steven, S. Kunze, M. K. Tauchert, K. Appel, M. Brechtel, I. Broehl, K. Fiedler, R. Geisler, S. M. Hopff, K. Knaub, C. Lee, S. Nunes de Miranda, S. Raquib, G. Sauer, M. Scherer, J. J. Vehreschild, P. Wagner, L. Wolf, J. C. Hellmuth, K. Guenther, F. Haug, J. Haug, A. Horn, M. Kohls, C. Fiessler, P. U. Heuschmann, O. Miljukov, C. Nuernberger, J. P. Reese, L. Schmidbauer, I. Chaplinskaya, S. Hanss, D. Krefting, C. Pape, M. Rainers, A. Schoneberg, N. Weinert, T. Bahls, W. Hoffmann, M. Nauck, C. Schaefer, M. Schattschneider, D. Stahl, H. Valentin, P. Heuschmann, A. L. Hofmann, S. Jiru‐Hillmann, J. P. Reese, S. Herold, P. Heuschmann, R. Heyder, W. Hoffmann, T. Illig, S. Schreiber, J. J. Vehreschild, M. Witzenrath

**Affiliations:** ^1^ Department of Neurology and Experimental Neurology Charité—Universitätsmedizin Berlin Berlin Germany; ^2^ Berlin School of Mind and Brain Humboldt‐Universität zu Berlin Berlin Germany; ^3^ Department of Infectious Disease, Respiratory Medicine and Critical Care Charité—Universitätsmedizin Berlin Berlin Germany; ^4^ Center for Stroke Research Berlin Berlin Germany; ^5^ German Center for Mental Health (DZPG), Partner Site Berlin Germany; ^6^ German Center for Neurodegenerative Diseases (DZNE), Partner Site Berlin Germany; ^7^ German Centre for Cardiovascular Research (DZHK), partner Site Berlin Germany; ^8^ Department of Anaesthesiology LMU University Hospital, LMU Munich Munich Germany; ^9^ Institute of Medical Data Science University Hospital Würzburg Würzburg Germany; ^10^ Institute of Clinical Epidemiology and Biometry University Würzburg Würzburg Germany; ^11^ Department I of Internal Medicine, Excellence Center for Medical Mycology (ECMM), Faculty of Medicine and University Hospital Cologne University of Cologne Cologne Germany; ^12^ Division of Clinical Immunology, Department I of Internal Medicine, Faculty of Medicine and University Hospital Cologne University of Cologne Cologne Germany; ^13^ Institute of Translational Research, Cologne Excellence Cluster on Cellular Stress Responses in Aging‐Associated Diseases (CECAD), Faculty of Medicine and University Hospital Cologne University of Cologne Cologne Germany; ^14^ Department I of Internal Medicine University Medical Center Schleswig‐Holstein Kiel Germany; ^15^ Deutsche Telekom Healthcare and Security Solutions GmbH Berlin Germany; ^16^ Department of Radiology LMU University Hospital, LMU Munich Munich Germany; ^17^ Comprehensive Pneumology Center (CPC‐M), member of the German Center for Lung Research (DZL) Munich Germany; ^18^ Department of Internal Medicine Justus‐Liebig‐University, Universities of Giessen and Marburg Lung Center, German Center for Lung Research Giessen Germany; ^19^ The Cardio‐Pulmonary Institute Giessen Germany; ^20^ Institute for Lung Health Giessen Germany; ^21^ Institute for Digital Medicine and Clinical Data Sciences, Faculty of Medicine Goethe University Frankfurt Frankfurt am Main Germany; ^22^ German Center for Lung Research (DZL) Berlin Germany

**Keywords:** cognitive dysfunction, COVID‐19, fatigue, magnetic resonance imaging, post‐acute COVID‐19 syndrome

## Abstract

**Objective:**

Neuropsychiatric symptoms are among the most prevalent sequelae of COVID‐19, particularly among hospitalized patients. Recent research has identified volumetric brain changes associated with COVID‐19. However, it currently remains poorly understood how brain changes relate to post‐COVID fatigue and cognitive deficits. We, therefore, aimed to assess structural brain changes after hospitalization for COVID‐19 and their associations with cognitive performance and fatigue.

**Methods:**

We analyzed data from *n* = 57 patients previously hospitalized for COVID‐19 (63% male, mean age 52 years) from the prospective, multicentric high‐resolution platform of the German National Pandemic Cohort Network (NAPKON‐HAP) and *n* = 57 matched healthy control participants (HC). We assessed cortical thickness and subcortical volumes in high‐resolution T1‐weighted MRI and their associations with cognitive performance (Montreal Cognitive Assessment) and fatigue (Fatigue Severity Scale).

**Results:**

Patients exhibited statistically significant reductions of cortical thickness in parahippocampal gyri and the temporal lobe (all *p*[FDR‐corrected] < 0.05) as well as reduced hippocampal volumes compared to HC (left, Cohen's *d* [95% CI] = 0.50 [0.12–0.8]; right *d* = 0.43 [0.05–0.80]). Higher acute COVID‐19 severity was associated with reduced cortical thickness, particularly in the olfactory system. Furthermore, reduced cortical thickness of the temporal poles and the anterior and posterior cingulate gyrus was associated with more severe post‐acute fatigue.

**Interpretation:**

Our results identify long‐lasting macrostructural brain changes after moderate to severe COVID‐19 that correlate with acute disease severity and long‐term fatigue. Early identification and targeted interventions for patients at risk of persistent brain changes are needed.

**Trial Registration:**

NAPKON‐HAP is registered at clinicaltrials.gov (NCT04747366)

## 1 Introduction

In the first year after infection with SARS‐CoV‐2, 21% of patients suffer from clinically relevant post‐viral fatigue and 23% experience cognitive deficits [1]. In patients who were hospitalized for COVID‐19, this prevalence is even higher, with up to 52% for fatigue, 70% for mild cognitive deficits, and 17% for moderate to severe cognitive deficits [2, 3, 4]. However, the mechanisms behind these neuropsychiatric symptoms and their relationship to long‐term structural brain changes remain incompletely understood [5]. Given the high prevalence and functional relevance of post‐COVID fatigue and cognitive impairment, identifying potential neural correlates of these symptoms has become a key research priority. Brain magnetic resonance imaging (MRI) can help to elucidate whether persistent symptoms reflect structural brain alterations or are primarily functional or systemic in nature.

Several studies have investigated volumetric brain changes in patients after COVID‐19 [6, 7, 8, 9, 10, 11, 12, 13, 14, 15, 16]. One of the most consistent findings across these studies is a reduction in gray matter volume and cortical thickness in olfaction‐related brain regions, especially the parahippocampal gyrus, orbitofrontal cortex, and insula [6, 7, 8]. Moreover, volume reductions in these regions were associated with anosmia [9]. Potential mechanisms of olfaction‐related volume loss include chronic inflammation, microvascular injury, secondary neuroplastic changes, as well as anterograde neurodegeneration from olfactory neurons [17].

However, few studies have investigated volumetric changes specifically in patients with post‐COVID fatigue or cognitive deficits. Recently, we observed that patients with mild to moderate COVID‐19 and post‐COVID fatigue exhibit reduced putamen volume and shape deformations of the putamen, pallidum and thalamus, which was associated the severity of fatigue and cognitive deficits [18]. Diez‐Cirarda et al. found that gray matter volumes in the parahippocampal area, superior temporal gyrus, and anterior cerebellar area were associated with deficits in attention and processing speed in post‐COVID patients on average 11 months after infection [6].

Although some studies included small subgroups of patients with severe COVID‐19 [6, 9], sufficiently powered volumetric analyses in hospitalized cohorts are scarce. Qin et al. found reduced cortical thickness in 32 previously hospitalized patients with severe COVID‐19 in the insula, and superior temporal gyrus as well as reduced volume of the hippocampus, putamen and thalamus compared to healthy control participants (HC) [8]. However, cognitive performance or symptoms of fatigue were not assessed.

In order to assess structural brain changes after hospitalization for COVID‐19 and their association with cognitive performance and fatigue, we analyzed data from the high‐resolution platform of the German National Pandemic Cohort Network (NAPKON‐HAP). Specifically, we hypothesized that olfactory and limbic areas would show reduced cortical thickness and that the hippocampus as a limbic structure closely connected with olfactory areas would show reduced volume. Gray matter atrophy in these areas was hypothesized to be associated with acute COVID‐19 disease severity and long‐term cognitive deficits. In addition, we hypothesized that reductions in cortical thickness as well as basal ganglia volumes would be associated with fatigue severity in these patients.

## 2 Methods

### 2.1 Study Design and Participants

NAPKON‐HAP is a prospective, longitudinal multicenter study in Germany, designed to comprehensively study organ‐specific sequelae of SARS‐CoV‐2 in hospitalized patients. A detailed description of the study has been published previously [19].

Eligibility criteria were (1) hospitalization for polymerase‐chain‐reaction‐confirmed COVID‐19 with typical clinical symptoms, (2) age 18 years or older; (3) written consent to participate in the study by the patient or an appropriate legal representative. Exclusion criteria comprised refusal to participate by the patient or legal representative or any condition preventing supplemental blood sampling.

For the current analysis, clinical data were collected longitudinally from patients during hospital treatment (3 times per week for up to 5 weeks or until discharge) at three study sites in Germany (Charité—Universitätsmedizin Berlin; LMU Klinikum München; Universitätsklinikum Gießen). After hospital discharge, structured follow‐up visits occurred over a period of up to 36 months from the onset of the first symptoms of COVID‐19. MRI and neuropsychological testing were performed at 3 months after discharge. Data collection took place from November 2020 to November 2022. Data from healthy control partici (HC) participants scanned at the Berlin study site were propensity‐score‐matched to these patients for sex and age using the R package *MatchIt* [20].

At the time of data analysis, *n* = 85 patients had received a follow‐up MRI. After exclusion of participants with missing T1‐weighted image (*n* = 11), severe MRI artifacts (*n* = 11), or post‐ischemic structural lesions (*n* = 6), volumetric analysis was performed in *n* = 57 patients. Patient sample characteristics are shown in Table 1. Pre‐infection neurological diseases comprised migraine (*n* = 1), multiple sclerosis (*n* = 1), myasthenia gravis (*n* = 1), transitory ischemic attack without residual symptoms or visible lesions on MRI (*n* = 1), chronic vertigo of unknown etiology (*n* = 1), and chronic pain syndrome (*n* = 1). HC participants were well matched by sex (m:f patients 36:21, HC 31:26; chi‐squared = 0.579, *p* = 0.447) and age (mean patients 52.4, HC 50.5; *t* = −0.76, *p* = 0.447).

**TABLE 1:** Patient sample characteristics (*N* = 57).

Characteristic	*n* /*N* (%) or *M* (SD)
*Sociodemographic*	
Sex at birth	
Male	36/57 (63%)
Female	21/57 (37%)
Age [years]	53 (13)
Years of education	14.7 (2.9)
*Lifestyle*	
BMI	28.2 (6.1)
Ever smoker	16/42 (38%)
*Pre‐infection comorbidity*	
Chronic neurological disease	6/55 (11%)
Any psychiatric disease	1/54 (2%)
Chronic lung disease	9/57 (16%)
Chronic kidney disease	6/55 (11%)
Chronic liver disease	1/55 (2%)
Cardiovascular disease	26/56 (46%)
Active cancer disease	2/57 (4%)
Chronic hematological disease	4/57 (7%)
Diabetes	8/55 (15%)
Rheumatological/immunological disease	4/55 (7%)
*Clinical*	
Vaccinated against SARS‐CoV‐2	23/49 (47%)
Acute COVID‐19 severity (WHO ordinal scale)	
4 – Hospitalized, no oxygen therapy	14/57 (25%)
5 – Oxygen by mask or nasal prongs	25/57 (44%)
6 – Oxygen by NIV or high flow	6/57 (11%)
7 – Intubation and mechanical ventilation, pO_2_/FiO_2_ ≥ 150 or SpO_2_/FiO_2_ ≥ 200	4/57 (7%)
8 – Mechanical ventilation pO_2_/FiO_2_ < 150 (SpO_2_/FiO_2_ < 200) or vasopressors	4/57 (7%)
9 – Mechanical ventilation pO_2_/FiO_2_ < 150 and vasopressors, dialysis, or ECMO	4/57 (7%)
Number of acute COVID‐19 symptoms	3.43 (1.76)

Abbreviations: BMI, body mass index; ECMO, extracorporeal membrane oxygenation; M, mean; NIV, noninvasive ventilation; SD, standard deviation.

### 2.2 Ethics and Study Registration

All participants provided written informed consent in accordance with the Declaration of Helsinki. The study was reviewed and approved by the Charité Ethics Committee in Berlin (EA2/066/20, EA2/226/21) as well as local ethics committees at each participating study center. NAPKON‐HAP is registered at clinicaltrials.gov (NCT04747366).

### 2.3 Imaging Measures

#### 2.3.1 MRI Data Acquisition

T1‐weighted structural MRI of the brain was acquired at 3 Tesla using MAGNETOM Prisma (Berlin, Munich) and Skyra (Gießen) scanners (Siemens, Erlangen, Germany) and a magnetization‐prepared rapid gradient echo (MPRAGE) sequence with the following parameters: field of view (FOV) 240 × 256 mm, 176 or 192 slices, voxel size 1 mm or 0.9 mm isotropic, repetition time (TR) 2500 ms or 2300 ms, echo time (TE) 2.64 ms or 2.32 ms, inversion time (TI) 1000 ms or 900 ms, flip angle 8 degrees.

#### 2.3.2 MRI Image Processing

T1‐weighted images were visually inspected for pathological features by two independent raters (TJH and FS). In instances of disagreement regarding the presence of relevant pathology, a third independent rater (CF) was consulted for a decisive evaluation. Scans exhibiting relevant macrostructural pathology, which could potentially influence volumetric analyses, were systematically excluded from the analysis (*n* = 6).

Images were preprocessed using FMRIB Software Library (FSL) robust field‐of‐view adjustment and reorient to standard. Following preprocessing, we employed an automated segmentation and parcellation process using FreeSurfer recon‐all (version 7.2). The resulting segmentations were visually inspected by FS. In cases where the quality of automated segmentations was questionable, further consultation with TJH and CF was sought (*n* = 4). Instances where automatic segmentation failed to yield accurate results were addressed through manual corrections using the FreeSurfer FreeView tool. However, scans that could not be satisfactorily corrected, particularly those marred by severe artifacts, were also excluded from further analysis (*n* = 11). Mean cortical thickness for all cortical parcels in the Destrieux atlas and volumes of subcortical structures were extracted from the FreeSurfer output.

### 2.4 Other Measures

#### 2.4.1 Cognitive Assessment

Cognitive performance was evaluated with the Montreal Cognitive Assessment (MoCA), an established assessment tool that generates scores between 0 (severe cognitive deficits) to 30 (no deficits). Following the guidelines of the test, an extra point was added to scores of participants who had fewer than 12 years of formal education. A score of 26 or higher was considered normal, while scores of 18–25 indicated mild, 10–17 moderate, and 9 or less severe cognitive impairments [21].

#### 2.4.2 Fatigue

The Fatigue Severity Scale (FSS) was employed to assess fatigue symptoms. This validated and widely‐used self‐report questionnaire consists of nine statements about the previous week, each rated on a Likert‐scale from 1 (strong disagreement) to 7 (strong agreement), where higher scores denote greater fatigue severity [22]. The overall score was calculated as the average of the nine item scores, providing a range between 1 and 7. A score of 1–3 is considered indicative of mild fatigue, 4–5 as moderate fatigue, and 6–7 as severe fatigue.

#### 2.4.3 Other Measures

Sociodemographic parameters, medical history and potential risk factors, current medication, assessment of clinical status, disease symptoms, and patient‐reported outcome measures (PROMIS) were collected during hospital stay. Data on disease severity as reflected by the WHO ordinal severity scale, concomitant medication, intercurrent diagnoses, and outcomes were collected daily during hospital stay.

### 2.5 Statistical Procedures

All statistical tests were two‐tailed and performed in R version 4.0.2. *p*‐values < 0.05 were considered statistically significant.

Differences between patients and HC participants were assessed using independent samples t‐tests. Effect sizes were quantified using Cohen's *d* including 95% confidence intervals. *p*‐values for cortical parcels (148 tests) and basal ganglia/thalamus (10 tests) were adjusted for multiple comparisons to control the false discovery rate (FDR). For brain regions with statistically significant group differences, we fitted separate multivariable linear regression models with sex, age, and cortical thickness as the independent variables and the respective outcome (MoCA total score or FSS total score) as the dependent variable. To assess associations between disease severity and cortical thickness, an ordinal regression model adjusted for sex and age was fitted using the *polr* function from the R package *MASS*.

Since previous reports suggested associations of basal ganglia and thalamus volumes with fatigue, cognition, and/or COVID‐19 disease severity, we explored such associations in the patient group. Associations between subcortical volumes and WHO scores did not show an ordinal distribution. Therefore, we dichotomized COVID‐19 severity into moderate (not requiring invasive ventilation, WHO scores 4–6) and severe (invasive ventilation, vasopressors, or ECMO, WHO scores 7–9).

### 2.6 Sensitivity Analyses

The analysis includes one patient who had a stroke and one patient with multiple sclerosis. Since these diseases can affect volumetric outcomes (even in the absence of visible lesions), we repeated all analyses after excluding these two patients. In addition, all models were repeated including MRI scanner type, study site, comorbidity, and body mass index (BMI) as covariates.

### 2.7 Role of the Funding Source

The funders were not involved in study design, data collection, data analysis, interpretation of data, writing of the report or decision to submit the paper for publication. TJH, FS and CF had access to the data and are finally responsible for the decision to submit the current work for publication.

## 3 Results

### 3.1 Reduced Cortical Thickness and Hippocampal Volumes

**FIGURE 1:**
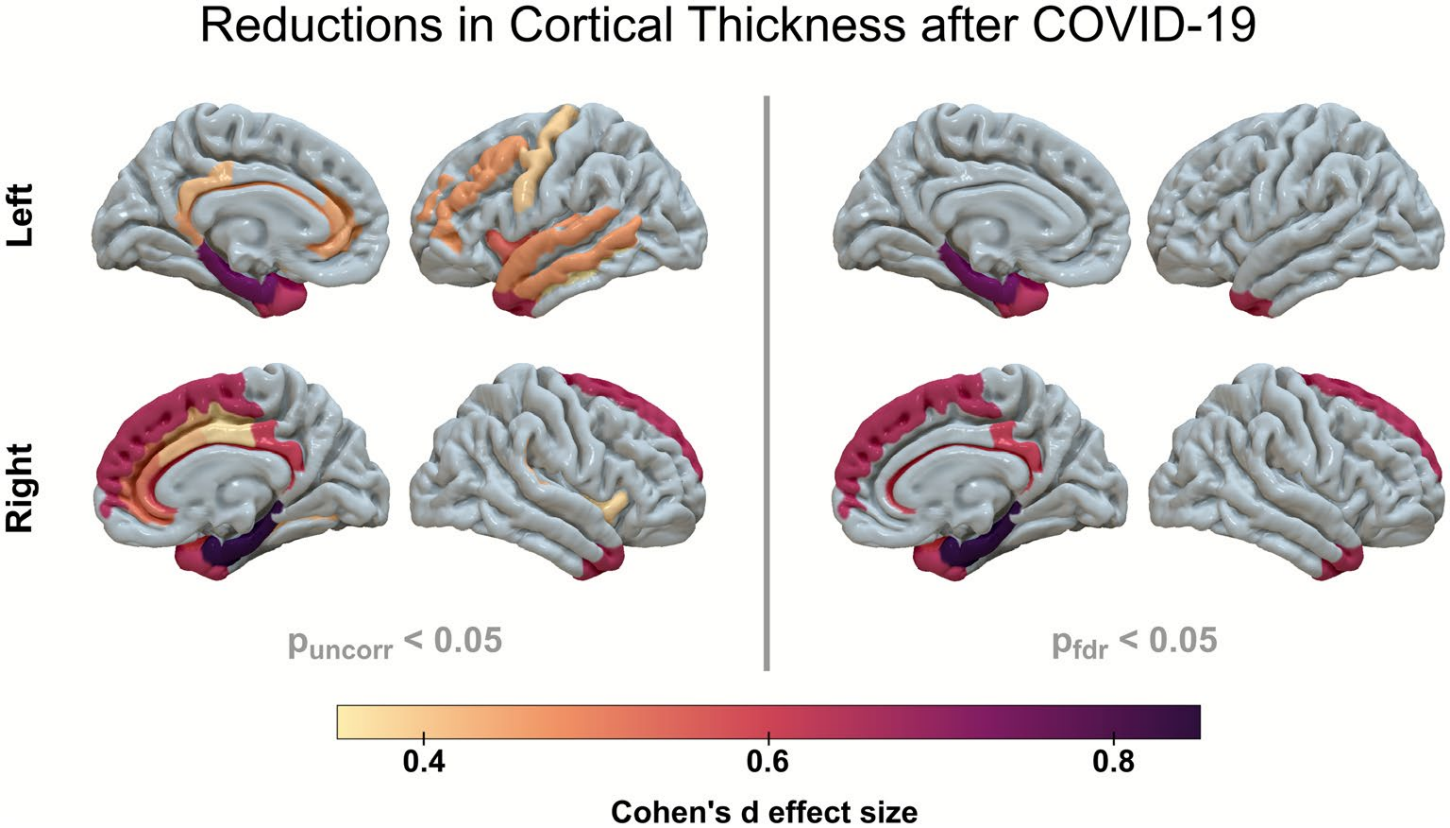
Regions with statistically significantly reduced cortical thickness in patients after hospitalization for COVID‐19 compared to matched healthy control participants. FDR, false discovery rate.

Patients hospitalized for COVID‐19 showed statistically significant reductions in cortical thickness compared to HC after 3 months (Figure 1), with the strongest effects in the parahippocampal gyri, the temporal poles, and the right frontal cortex (see Table S1 for comprehensive statistics).

**FIGURE 2:**
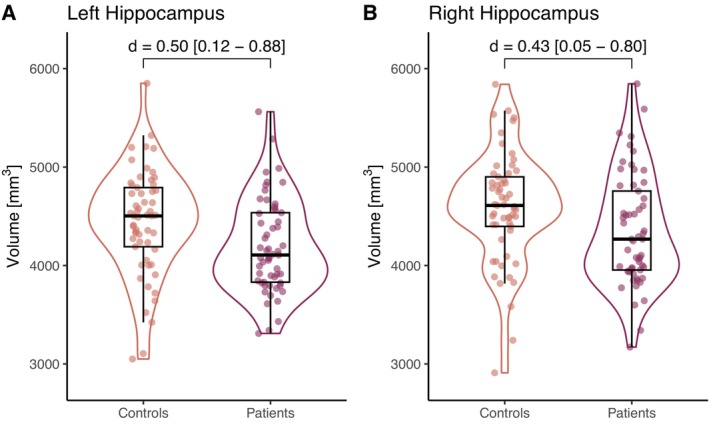
Patients after hospitalization for COVID‐19 show statistically significantly lower volumes of the left (A) and right (B) hippocampus compared to healthy control participants. Effect size as Cohen's *d* [95% confidence interval].

Furthermore, patients showed significantly lower volumes of the left (*d* = 0.50, *p* = 0.009) and right (*d* = 0.43, *p* = 0.020) hippocampus compared to HC (Figure 2). Among the basal ganglia, the left pallidum showed a volume reduction in patients (*d* = 0.46, *p* = 0.017), which was limited to statistical tendency (*p*_*FDR*_ = 0.166) after correction for multiple testing (Table S2).

### 3.2 Associations With Acute COVID‐19 Severity and Post‐Acute Fatigue

In this cohort, 79% (45/57) of patients were moderately affected during acute COVID‐19, requiring no or noninvasive oxygen support (WHO categories 4–6), while 21% (12/57) had a severe disease course requiring invasive ventilation, vasopressors, or organ support (WHO categories 7–9, Table 1). At the time of the MRI scan, 40% (16/40) of patients showed cognitive deficits according to the MoCA total score (37% mild, 3% moderate, none severe). Relevant fatigue according to the FSS was reported by 55% (23/42) of patients (40% moderate and 15% severe).

**FIGURE 3:**
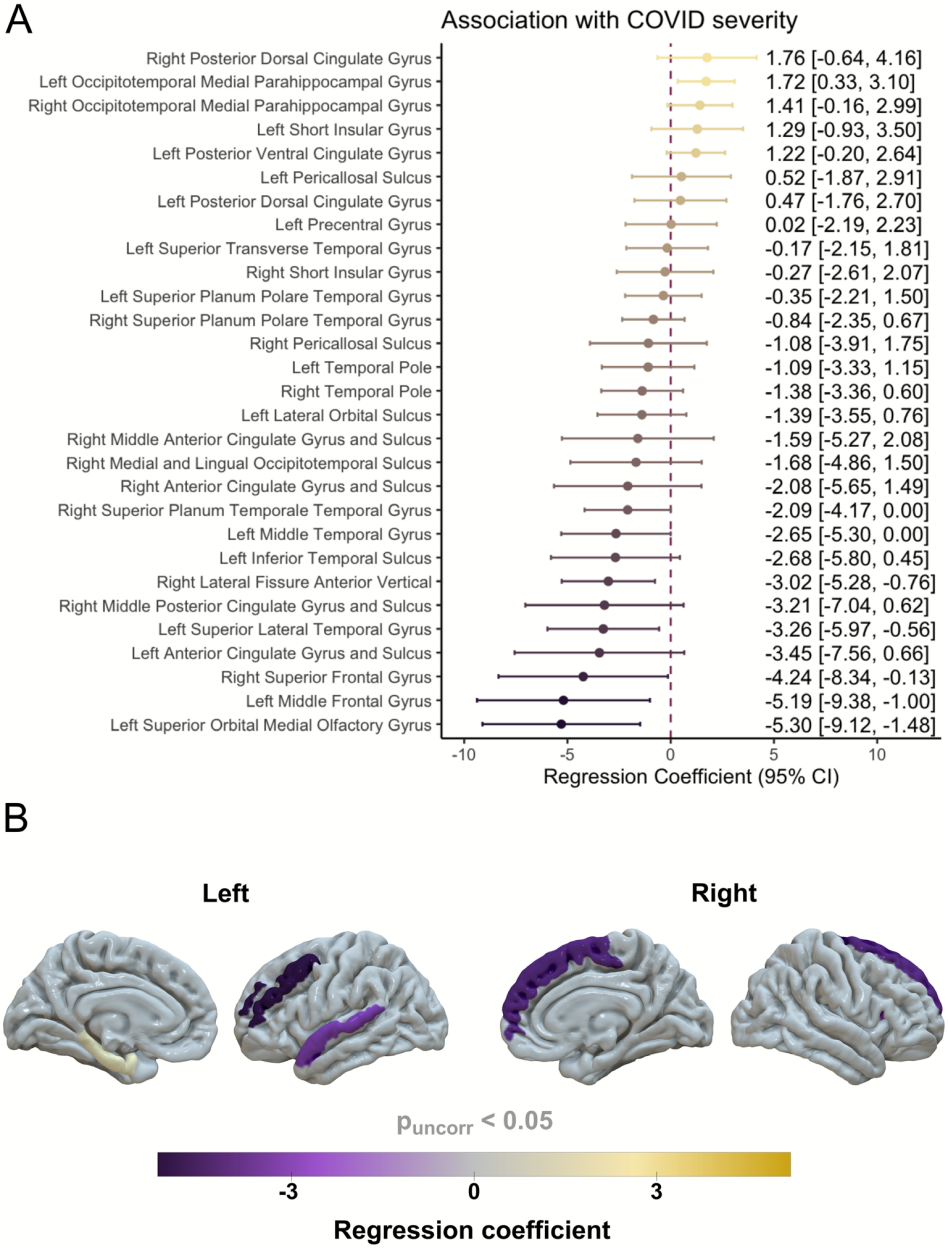
Association between acute COVID‐19 severity (WHO Ordinal Scale) and reduced (purple) or increased (yellow) cortical thickness in brain regions with between‐group differences (Figure 1), after adjustment for sex and age. (A) Regression coefficients and 95% confidence intervals, (B) effect localization in the brain.

Acute COVID‐19 severity (as measured by the WHO Ordinal Scale) was associated with reduced cortical thickness in the left medial orbital sulcus adjacent to the olfactory area (*p* = 0.007), the right anterior segment of the vertical lateral fissure (*p* = 0.009), the parahippocampal part of the left medial occipito‐temporal gyrus (*p* = 0.015), the left middle frontal gyrus (*p* = 0.015), the left superior lateral temporal gyrus (*p* = 0.018), and the right superior frontal gyrus (*p* = 0.043), after adjustment for sex and age (Figure 3). COVID‐19 severity was also associated with increased cortical thickness in the left occipitotemporal medial parahippocampal gyrus (*p* = 0.015).

**FIGURE 4:**
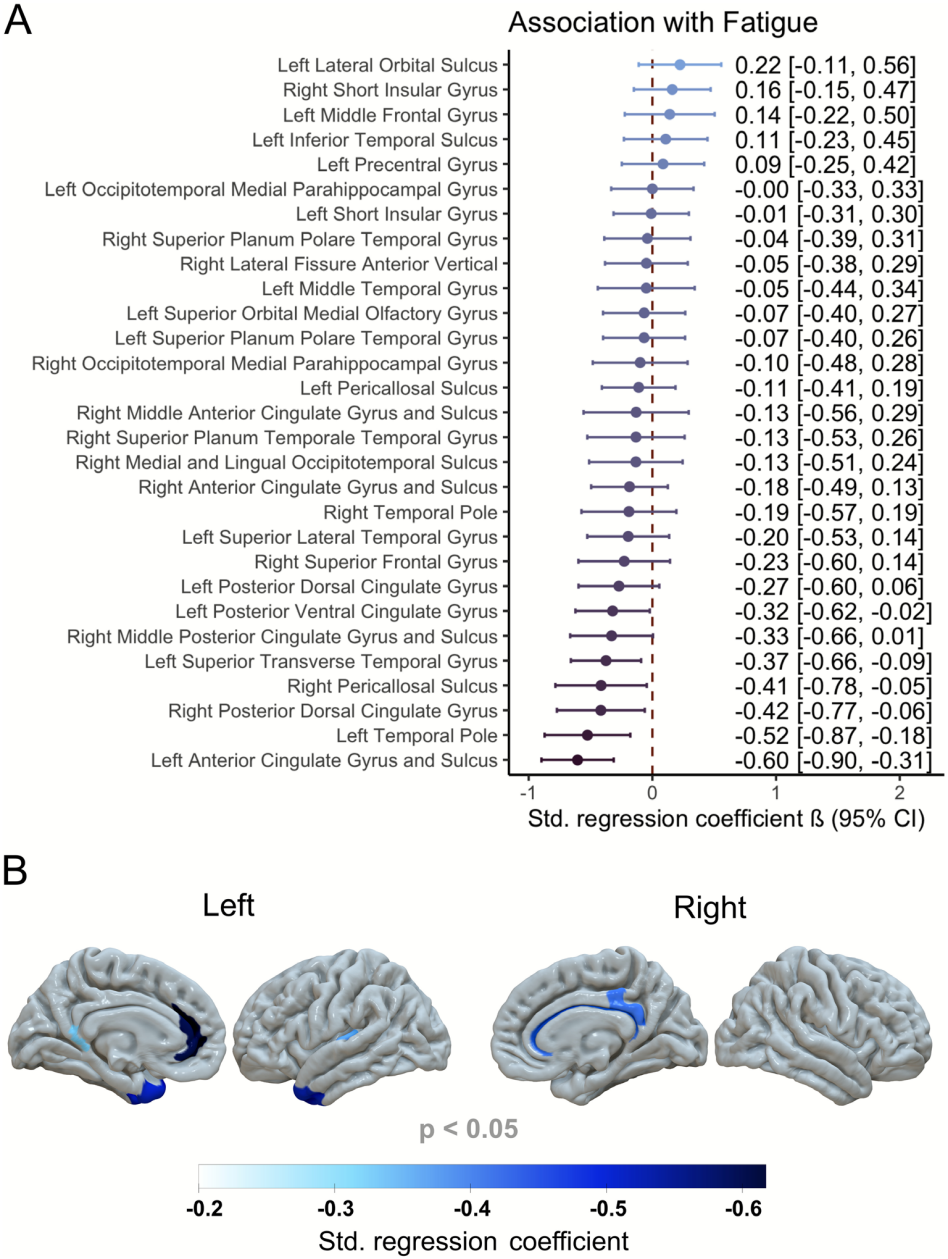
Association between post‐COVID fatigue severity (FSS total score) and cortical thickness in brain regions with between‐group differences (Figure 1), after adjustment for sex and age: (A) Standardized regression coefficients and 95% confidence intervals, (B) effect localization in the brain.

Furthermore, post‐acute fatigue severity was associated with reduced cortical thickness in the left anterior cingulate gyrus and sulcus (*p* < 0.001), left temporal pole (*p* = 0.005), left superior transverse temporal gyrus (*p* = 0.014), right posterior dorsal cingulate gyrus (*p* = 0.027), right pericallosal sulcus (*p* = 0.034), and left posterior ventral cingulate gyrus (*p* = 0.043), after adjustment for sex and age (Figure 4).

There was no statistically significant association between cortical thickness and cognitive performance after adjustment for sex, age and education (Table S3).

### 3.3 Association Between Subcortical Volumes, COVID‐19 Severity, Fatigue, and Cognitive Deficits

**FIGURE 5:**
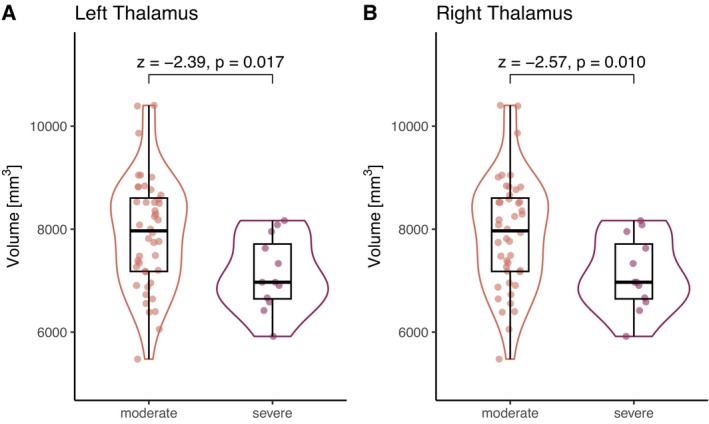
Patients with severe acute COVID‐19 (invasive ventilation, WHO Score 7–9) showed statistically significantly lower volumes of the left (A) and right (B) thalamus compared to patients with moderate disease severity (hospitalized without invasive ventilation, WHO Score 4–6). *Z*‐ and *p*‐values obtained from logistic regression models adjusted for sex and age.

After adjusting for sex and age, higher acute COVID‐19 severity was statistically significantly associated with lower volumes in the left and right thalamus (Figure 5). There were no other statistically significant associations between subcortical volumes and acute COVID‐19 severity, post‐acute fatigue, or cognitive performance (Tables S9–S11).

### 3.4 Sensitivity Analyses

Excluding one patient who had a stroke and one patient with multiple sclerosis led to larger effect sizes in analyses involving subcortical structures and smaller effect sizes regarding cortical thickness but had no relevant effects on the main results. Adding scanner type, study site, somatic comorbidity, or body‐mass index (BMI) as additional covariates in the regression models did not alter the overall pattern of results (Tables S3–S11). For the association between cortical thickness and cognitive performance (MoCA), adjustment for site, comorbidity, and BMI slightly increased effect sizes, with significant associations emerging in the left parahippocampal gyrus and in the left medial orbital–olfactory sulcus (Table S3). Associations with fatigue (FSS) were largely robust across models; inclusion of study site yielded one additional significant region in the right mid‐posterior cingulate cortex, whereas adjustment for comorbidity or BMI reduced effect sizes, leaving only the left anterior cingulate cortex significant (Table S4). For disease severity (WHO score), associations with cortical thickness were also consistent, although inclusion of study site or comorbidity led to slightly larger effect sizes and additional significant temporal regions (left temporal pole, left middle temporal gyrus, left planum polare, and right planum temporale), while the left parahippocampal gyrus no longer reached significance after site adjustment. Adjustment for BMI produced a comparable pattern but with regional shifts, such that effects in the left medial orbital and olfactory cortex, left lateral superior temporal gyrus, and right superior frontal gyrus were no longer significant, while new associations emerged in the left anterior cingulate and left short insular gyri (Table S5). In the hippocampal and basal ganglia analyses, the inclusion of scanner type, site, comorbidity, or BMI did not change the findings, and no new significant associations emerged (Tables S6–S11). The negative association between thalamic volume and COVID‐19 disease severity remained stable across all sensitivity analyses, although BMI adjustment slightly reduced the effect size, leaving only the right thalamus significant (Table S11).

Patients with missing data on the FSS or MoCA did not significantly differ from patients with complete data in terms of available baseline characteristics (sex, age, education and comorbidity; Tables S12 and S13).

## 4 Discussion

In this prospective multicenter study, patients who had been hospitalized for COVID‐19 showed statistically significant reductions in cortical thickness in parahippocampal areas and the temporal lobe as well as lower hippocampal volumes compared to HC participants. Moreover, higher acute COVID‐19 severity was associated with reduced cortical thickness 3 months after discharge, particularly in regions adjacent to olfactory areas and in the frontal and temporal cortices. Additionally, 40% of patients experienced cognitive deficits and 55% reported relevant fatigue. Importantly, post‐acute fatigue severity was associated with reduced cortical thickness in the cingulate gyrus and the temporal lobe. Our results show that patients with moderate to severe COVID‐19 exhibit long‐lasting macrostructural brain changes that are associated with acute disease severity and post‐acute fatigue severity.

The most pronounced cortical thinning was observed in the temporal lobes, and particularly parahippocampal areas. This is in line with previous studies which consistently reported volume and thickness reductions in these olfaction‐related brain areas [6, 7, 8]. Our finding of reduced hippocampal volumes could be regarded as complementary to these findings, since the hippocampus is a key region for olfactory processing and has previously been found to be affected in COVID‐19 [8]. However, most studies only contained small subgroups of hospitalized patients and results were heterogeneous with regard to localization and direction of brain changes [23]. Recently, it has been suggested that structural and metabolic changes in these regions could result from a combination of deafferentation, neuroinflammation, and reorganization of neural circuits due to the lack of olfactory input [24].

Importantly, post‐acute fatigue severity was associated with reductions in cortical thickness, which was most pronounced in the anterior and posterior cingulate gyrus and sulcus. To our knowledge, this association has not yet been shown in the context of SARS‐CoV‐2 infection. The anterior cingulate cortex is involved in the perception of effort, and structural as well as functional changes in this region are associated with exercise‐induced fatigue [25]. Furthermore, functional changes in the posterior cingulate cortex are also associated with fatigue symptoms in patients with multiple sclerosis [26]. These findings potentially point to shared mechanisms, which may include neuroinflammation triggered by systemic (auto)immune responses, cytotoxic immune reactions, and reactivation of latent viruses [27]. Localized cortical thinning could be considered a neural correlate of post‐COVID fatigue, and cortical thickness may thus serve as a predictive imaging marker. Future longitudinal studies should assess whether reductions in cortical thickness can predict long‐term trajectories of fatigue severity in patients who were previously hospitalized for COVID‐19.

In our primary analyses, we observed no significant associations between cognitive performance and cortical thickness or subcortical deep gray matter volumes. However, in sensitivity analyses adjusting for site and somatic comorbidities, a trend towards a thinner parahippocampal cortex being associated with lower MoCA scores became statistically significant. On the one hand, the effect size of this association was relatively small, requiring large a large sample size for detection. On the other hand, the MoCA as a brief cognitive screening tool might not be sufficiently sensitive to detect subtle or domain‐specific cognitive impairments. Importantly, the observed pattern is consistent with previous studies finding associations between parahippocampal cortical thinning and cognitive deficits in patients with post‐COVID syndrome [6, 28, 29]. Future studies with larger samples and more comprehensive cognitive assessments are warranted to clarify how structural alterations of the parahippocampal cortex are associated with post‐COVID cognitive outcomes.

Early identification of patients at risk for long‐term brain changes could allow for the implementation of targeted intervention and rehabilitation programs. Our results clearly suggest that patients hospitalized with moderate to severe COVID‐19 should be closely monitored for cognitive deficits and fatigue during the post‐COVID phase. It is likely that fatigue will persist for several months to years in many patients and will require targeted clinical management strategies. To this end, pharmacological and non‐pharmacological interventions should be explored [30].

In an exploratory approach, we furthermore found that patients with severe COVID‐19 requiring invasive ventilation had statistically significantly lower thalamus volumes than those requiring no or noninvasive oxygen support. This is consistent with a study by Qin et al. who also found severity‐dependent reductions in thalamus volume compared to HC [8]. Furthermore, changes in shape and microstructural integrity of the thalamus were previously found in patients with mild COVID‐19 compared to HC participants [18]. While the exact mechanisms of severity‐dependent thalamic volume reductions are insufficiently understood, it has been suggested that the thalamus is highly sensitive to low oxygen levels and thus particularly vulnerable to acute necrotizing encephalopathy from late‐stage immune demyelination in COVID‐19 [8, 31]. Therefore, preventing hypoxia might be especially relevant for preserving thalamic integrity and function.

Contrary to our main hypothesis, we observed that greater COVID‐19 severity was associated with greater cortical thickness in the left parahippocampal region. This observation might represent a spurious result arising from statistical variability or could reflect segmentation or registration artifacts. Alternatively, a compensatory or reactive process might underlie cortical thickening, such as gliosis, glial hypertrophy, or neuroinflammation that transiently increase apparent cortical thickness before atrophy ensues. Indeed, some studies in patients with long‐COVID reported increases in cortical thickness in various brain regions. For example, Besteher et al. found a progressive increase in cortical thickness in several brain regions, including the parahippocampal gyrus in long‐COVID patients [28]. Similarly, Pacheco‐Jaime et al. reported increased thickness in frontal areas in a post‐COVID cohort compared to controls [32]. These observations suggest that structural “thickening” might occur in the subacute or intermediate phases of disease, potentially reflecting reactive gliosis or extracellular fluid shifts, before degenerative thinning dominates over longer time scales.

For these reasons, future studies should pursue longitudinal designs including pre‐infection baseline imaging, complement anatomical sequences with microstructural imaging modalities such as diffusion or quantitative MRI to better assess white matter tissue properties, use detailed neuropsychological assessments and recruit larger control cohorts with detailed cardiovaskular risk stratification and neuropsychiatric phenotyping.

### 4.1 Limitations and Strengths

Our results should be interpreted in light of the following limitations: While the overall study design is prospective and longitudinal, volumetric MRI analyses were cross‐sectional and do not allow conclusions about the temporal course, causal relations or the effect of structural brain abnormalities before SARS‐CoV‐2 infection. Intensive care treatment itself may have had effects on regional brain volumes that cannot be fully disentangled from disease‐specific effects in patients with severe COVID‐19. Detailed data on education, BMI and cardiovascular risk factors were not available for HC participants such that matching for these variables was not possible. In addition, MoCA and FSS data were not available in the HC group, such that group comparisons of fatigue and cognitive deficits were not possible, limiting inferences about the extent to which the observed associations are COVID‐specific. As the MoCA is a screening instrument, subtle cognitive deficits may have gone undetected that could have been identified with a comprehensive neuropsychological assessment. Several comorbidities present in the patient sample may impact brain macrostructure and cognitive performance. However, sensitivity analyses showed no relevant changes in the overall pattern of results after adjusting for comorbidity.

Strengths of the study include the prospective and multi‐center design, the representative cohort in terms of sociodemographics and comorbidity, the use of validated instruments to assess fatigue and cognitive performance as well as state‐of‐the‐art volumetric analyses of high‐resolution structural MRI.

## 5 Conclusion

This prospective multicenter study highlights macrostructural brain changes in patients after hospitalization for moderate to severe COVID‐19, especially in areas of the olfactory system. Importantly, these changes were associated with both acute disease severity and post‐acute fatigue. Specifically, our study identifies cortical thinning of the cingulate gyrus as a novel structural correlate of post‐COVID fatigue severity. These insights underscore the importance of early identification of patients at risk for long‐term brain changes and the need for targeted interventions to mitigate long‐lasting impairments.

## Author Contributions

**Tim J. Hartung, Florentin Steigerwald, Janne Vehreschild, and Carsten Finke:** conceptualization. **Tim J. Hartung, Florentin Steigerwald, and Jens Schaller:** data curation. **Tim J. Hartung and Florentin Steigerwald:** formal analysis. **Janne Vehreschild and Carsten Finke:** funding acquisition. **Peter Heuschmann, Philipp Koehler, Daniel Pape, Sophia Stöcklein, Martin Witzenrath, and Thomas Zoller:** investigation. **Tim J. Hartung, Florentin Steigerwald, Peter Heuschmann, Stephan Krohn, Sophia Stöcklein, Janne Vehreschild, and Carsten Finke:** methodology. **Cathrin Kodde, Jens Schaller, Sophia Stöcklein, Janne Vehreschild, and Carsten Finke:** project administration. **Sandra Frank, Philipp Koehler, Daniel Pape, Istvan Vadasz, Martin Witzenrath, Thomas Zoller, and Carsten Finke:** resources. **Carsten Finke:** supervision. **Tim J. Hartung and Amy Romanello:** visualization. **Tim J. Hartung:** writing – original draft. **Tim J. Hartung, Florentin Steigerwald, Amy Romanello, Cathrin Kodde, Matthias Endres, Sandra Frank, Peter Heuschmann, Philipp Koehler, Stephan Krohn, Daniel Pape, Jens Schaller, Sophia Stöcklein, Istvan Vadasz, Janne Vehreschild, Martin Witzenrath, Thomas Zoller, and Carsten Finke:** writing – review and editing.

## Conflicts of Interest

P.K. reports grants or contracts from German Federal Ministry of Research and Education (BMBF) B‐FAST (Bundesweites Forschungsnetz Angewandte Surveillance und Testung) and NAPKON (Nationales Pandemie Kohorten Netz, German National Pandemic Cohort Network) of the Network University Medicine (NUM), the State of North Rhine‐Westphalia and the Dr. Heinz Lux‐Stiftung; Consulting fees Ambu GmbH, Gilead Sciences, infill healthcare communication GmbH, Mundipharma Research Limited, Noxxon N.V. and Pfizer Pharma; Honoraria for lectures from Akademie für Infektionsmedizin e.V., Ambu GmbH, Astellas Pharma, BioRad Laboratories Inc., Datamed GmbH, European Confederation of Medical Mycology, Gilead Sciences, GPR Academy Ruesselsheim, HELIOS Kliniken GmbH, Jazz Pharmaceuticals Germany GmbH, Lahn‐Dill‐Kliniken GmbH, medupdate GmbH, MedMedia GmbH, MSD Sharp & Dohme GmbH, Pfizer Pharma GmbH, Sanofi‐Aventis Deutschland GmbH, Scilink Comunicación Científica SC, streamedup! GmbH, University Hospital and LMU Munich and VITIS GmbH; Participation on an Advisory Board from Ambu GmbH, Gilead Sciences, Mundipharma Research Limited and Pfizer Pharma; A filed patent at the German Patent and Trade Mark Office (DE 102021 113007.7); Other non‐financial interests from Elsevier, Wiley and Taylor & Francis online outside the submitted work. The other authors have reported no relevant conflicts of interest.

## Supporting information


**Data S1:** acn370260‐sup‐0001‐SupplementaryMaterials.pdf.

## Data Availability

All data of this study may be shared upon request to the NAPKON data use and access committee. Interested parties can access relevant data governance information and submit their research proposal to the NAPKON use and access committee at https://proskive.napkon.de.
