# Supplementary material for: Post‐COVID Fatigue Is Associated With Reduced Cortical Thickness After Hospitalization
Source: Ann Clin Transl Neurol. 2025 Nov 25;13(4):755–66. doi: 10.1002/acn3.70260 (PMC13071101; doi:10.1002/acn3.70260)
Supplement: Supplementary file 1 — Data S1: acn370260‐sup‐0001‐SupplementaryMaterials.pdf. [file ACN3-13-755-s001.pdf]

Supplementary Materials

Post-COVID fatigue is associated with reduced cortical thickness after hospitalization

Running title: COVID-19 and gray matter reduction

Tim J. Hartung, MD MA[1], Florentin Steigerwald, BA[1], Amy Romanello, MSc[1,2], Cathrin Kodde, MD[3], Matthias Endres, MD[1,4,5,6,7], Sandra Frank, MD[8], Peter Heuschmann, MD MPH[9,10], Philipp Koehler, MD[11,12,13], Stephan Krohn, MD MSc[1,2], Daniel Pape, MD[14], Jens Schaller[15], Sophia Stöcklein, MD[16,17], Istvan Vadasz, MD PhD[18,19,20], Janne Vehreschild, MD[21], Martin Witzenrath, MD[3,22], Thomas Zoller, MD[3], Carsten Finke, MD[1,2] on behalf of the NAPKON Study Group

[1] Charité – Universitätsmedizin Berlin, Department of Neurology and Experimental Neurology, Berlin, Germany
[2] Humboldt-Universität zu Berlin, Berlin School of Mind and Brain, Berlin, Germany
[3] Charité – Universitätsmedizin Berlin, Department of Infectious Disease, Respiratory Medicine and Critical Care, Berlin, Germany
[4] Center for Stroke Research Berlin, Berlin, Germany
[5] German Center for Mental Health (DZPG), partner site Berlin
[6] German Center for Neurodegenerative Diseases (DZNE), partner site Berlin
[7] German Centre for Cardiovascular Research (DZHK), partner site Berlin
[8] Department of Anaesthesiology, LMU University Hospital, LMU Munich, Germany
[9] Institute of Medical Data Science, University Hospital Würzburg, Germany
[10] Institute of Clinical Epidemiology and Biometry, University Würzburg, Germany
[11] University of Cologne, Faculty of Medicine and University Hospital Cologne, Department I of Internal Medicine, Excellence Center for Medical Mycology (ECMM), Cologne, Germany
[12] University of Cologne, Faculty of Medicine and University Hospital Cologne, Department I of Internal Medicine, Division of Clinical Immunology, Cologne, Germany
[13] University of Cologne, Faculty of Medicine and University Hospital Cologne, Institute of Translational Research, Cologne Excellence Cluster on Cellular Stress Responses in Aging-Associated Diseases (CECAD), Cologne, Germany
[14] Department I of Internal Medicine, University Medical Center Schleswig-Holstein, Campus Kiel, Germany
[15] Deutsche Telekom Healthcare and Security Solutions GmbH, Berlin, Germany
[16] Department of Radiology, LMU University Hospital, LMU Munich, Germany
[17] Comprehensive Pneumology Center (CPC-M), member of the German Center for Lung Research (DZL), Munich, Germany
[18] Department of Internal Medicine, Justus-Liebig-University, Universities of Giessen and Marburg Lung Center, German Center for Lung Research, Giessen, Germany
[19] The Cardio-Pulmonary Institute, Giessen, Germany
[20] Institute for Lung Health, Giessen, Germany
[21] Goethe University Frankfurt, Faculty of Medicine, Institute for Digital Medicine and Clinical Data Sciences, Frankfurt am Main, Germany
[22] German Center for Lung Research (DZL), Berlin, Germany

Contents

Table S1. Group differences in cortical thickness between patients after COVID-19 and healthy control participants.......... 5

Table S2. Group differences in subcortical volumes between patients after COVID-19 and healthy control participants.......... 9

Table S3. Associations between cortical thickness and cognitive performance in patients after COVID-19, adjusted for sex, age, and years of education. *Abbreviations: lh - left hemisphere, rh - right hemisphere, S - sulcus, G - gyrus*.......... 10

Table S3a. Associations between cortical thickness and cognitive performance in patients after COVID-19, adjusted for sex, age, years of education and scanner type. *Abbreviations: lh - left hemisphere, rh - right hemisphere, S - sulcus, G - gyrus*.......... 11

Table S3b. Associations between cortical thickness and cognitive performance in patients after COVID-19, adjusted for sex, age, years of education and study site. *Abbreviations: lh - left hemisphere, rh - right hemisphere, S - sulcus, G - gyrus*.......... 12

Table S3c. Associations between cortical thickness and cognitive performance in patients after COVID-19, adjusted for sex, age, years of education and comorbidity. *Abbreviations: lh - left hemisphere, rh - right hemisphere, S - sulcus, G - gyrus*.......... 13

Table S3d. Associations between cortical thickness and cognitive performance in patients after COVID-19, adjusted for sex, age, years of education and body mass index. *Abbreviations: lh - left hemisphere, rh - right hemisphere, S - sulcus, G - gyrus*.......... 14

Table S4a. Associations between cortical thickness and fatigue severity in patients after COVID-19, adjusted for sex, age, and scanner type. *Abbreviations: lh - left hemisphere, rh - right hemisphere, S - sulcus, G - gyrus*.......... 15

Table S4b. Associations between cortical thickness and fatigue severity in patients after COVID-19, adjusted for sex, age, and study site. *Abbreviations: lh - left hemisphere, rh - right hemisphere, S - sulcus, G - gyrus*.......... 16

Table S4c. Associations between cortical thickness and fatigue severity in patients after COVID-19, adjusted for sex, age, and comorbidity. *Abbreviations: lh - left hemisphere, rh - right hemisphere, S - sulcus, G - gyrus*.......... 17

Table S4d. Associations between cortical thickness and fatigue severity in patients after COVID-19, adjusted for sex, age, and body mass index. *Abbreviations: lh - left hemisphere, rh - right hemisphere, S - sulcus, G - gyrus*.......... 18

Table S5a. Associations between cortical thickness and COVID-19 severity (WHO ordinal scale) in patients after COVID-19, adjusted for sex, age, and scanner type. *Abbreviations: lh - left hemisphere, rh - right hemisphere, S - sulcus, G - gyrus*.......... 19

Table S5b. Associations between cortical thickness and COVID-19 severity (WHO ordinal scale) in patients after COVID-19, adjusted for sex, age, and study site. *Abbreviations: lh - left hemisphere, rh - right hemisphere, S - sulcus, G - gyrus*.......... 20

Table S5c. Associations between cortical thickness and COVID-19 severity (WHO ordinal scale) in patients after COVID-19, adjusted for sex, age, and comorbidity. *Abbreviations: lh - left hemisphere, rh - right hemisphere, S - sulcus, G - gyrus*.......... 21

Table S5d. Associations between cortical thickness and COVID-19 severity (WHO ordinal scale) in patients after COVID-19, adjusted for sex, age, and body mass index. *Abbreviations: lh - left hemisphere, rh - right hemisphere, S - sulcus, G - gyrus.* 22
Table S6. Associations between hippocampus volumes and fatigue severity in patients with COVID-19. 23
Table S7. Associations between hippocampus volumes and cognitive performance, in patients with COVID-19. 23
Table S8. Associations between hippocampus volumes and COVID-19 severity (WHO Ordinal Scale 4-6 [no invasive ventilation] vs. 7-9 [invasive ventilation]). 24
Table S9. Associations between subcortical volumes and fatigue severity in patients after COVID-19, adjusted for sex and age. 24
Table S9a. Associations between subcortical volumes and fatigue severity in patients after COVID-19, adjusted for sex, age and scanner type. 24
Table S9b. Associations between subcortical volumes and fatigue severity in patients after COVID-19, adjusted for sex, age and study site. 25
Table S9c. Associations between subcortical volumes and fatigue severity in patients after COVID-19, adjusted for sex, age and comorbidity. 25
Table S9d. Associations between subcortical volumes and fatigue severity in patients after COVID-19, adjusted for sex, age and body mass index. 25
Table S10. Associations between subcortical volumes and cognitive performance in patients after COVID-19, adjusted for sex, age, and years of education. 26
Table S10a. Associations between subcortical volumes and cognitive performance in patients after COVID-19, adjusted for sex, age, years of education and scanner type. 26
Table S10b. Associations between subcortical volumes and cognitive performance in patients after COVID-19, adjusted for sex, age, years of education and study site. 26
Table S10c. Associations between subcortical volumes and cognitive performance in patients after COVID-19, adjusted for sex, age, years of education and comorbidity. 27
Table S10d. Associations between subcortical volumes and cognitive performance in patients after COVID-19, adjusted for sex, age, years of education and body mass index. 27
Table S11. Associations between subcortical volumes and COVID-19 severity (WHO Ordinal Scale 4-6 [no invasive ventilation] vs. 7-9 [invasive ventilation]), adjusted for sex and age. 28
Table S11a. Associations between subcortical volumes and COVID-19 severity (WHO Ordinal Scale 4-6 [no invasive ventilation] vs. 7-9 [invasive ventilation]), adjusted for sex, age and scanner type. 28
Table S11b. Associations between subcortical volumes and COVID-19 severity (WHO Ordinal Scale 4-6 [no invasive ventilation] vs. 7-9 [invasive ventilation]), adjusted for sex, age and study site. 28
Table S11c. Associations between subcortical volumes and COVID-19 severity (WHO Ordinal Scale 4-6 [no invasive ventilation] vs. 7-9 [invasive ventilation]), adjusted for sex, age and comorbidity. 29

Table S11d. Associations between subcortical volumes and COVID-19 severity (WHO Ordinal Scale 4-6 [no invasive ventilation] vs. 7-9 [invasive ventilation]), adjusted for sex, age and body mass index.. 29
Table S12. Baseline characteristics of patients with missing and complete Fatigue Severity Scale (FSS)... 30
Table S13. Baseline characteristics of patients with missing and complete Montreal Cognitive Assessment (MoCA)... 30

Table S1. Group differences in cortical thickness between patients after COVID-19 and healthy control participants.

Region	Cohen's d (95% CI)	p	p (FDR)
rh_G_oc_temp_med_Parahip	0.84 (0.46, 1.23)	0.000	0.003
lh_G_oc_temp_med_Parahip	0.75 (0.37, 1.13)	0.000	0.011
lh_G_temp_sup_Plan_polar	0.68 (0.29, 1.06)	0.000	0.023
lh_Pole_temporal	0.64 (0.26, 1.02)	0.001	0.024
rh_Pole_temporal	0.64 (0.26, 1.02)	0.001	0.024
rh_G_front_sup	0.64 (0.26, 1.02)	0.001	0.024
rh_G_cingul_Post_dorsal	0.60 (0.22, 0.98)	0.002	0.038
rh_S_pericallosal	0.59 (0.21, 0.97)	0.002	0.042
rh_G_temp_sup_Plan_polar	0.57 (0.19, 0.95)	0.003	0.049
lh_G_insular_short	0.54 (0.16, 0.92)	0.005	0.070
rh_G_and_S_cingul_Ant	0.52 (0.14, 0.90)	0.007	0.090
lh_S_orbital_lateral	0.47 (0.09, 0.84)	0.014	0.124
lh_G_front_middle	0.47 (0.09, 0.85)	0.014	0.124
lh_G_temporal_middle	0.47 (0.10, 0.85)	0.013	0.124
lh_S_pericallosal	0.47 (0.10, 0.85)	0.013	0.124
lh_G_temp_sup_Lateral	0.48 (0.10, 0.85)	0.013	0.124
lh_G_temp_sup_G_T_transv	0.48 (0.10, 0.86)	0.012	0.124
lh_G_cingul_Post_ventral	0.46 (0.08, 0.84)	0.016	0.131
lh_G_and_S_cingul_Ant	0.45 (0.07, 0.83)	0.018	0.141
rh_G_temp_sup_Plan_tempo	0.44 (0.07, 0.82)	0.020	0.151
rh_S_oc_temp_med_and_Lingual	0.43 (0.05, 0.80)	0.025	0.163
lh_G_cingul_Post_dorsal	0.43 (0.05, 0.80)	0.025	0.163
rh_G_and_S_cingul_Mid_Ant	0.43 (0.05, 0.80)	0.024	0.163
lh_G_precentral	0.41 (0.03, 0.78)	0.032	0.198
rh_Lat_Fis_ant_Vertical	-0.40 (-0.77, -0.02)	0.036	0.207
lh_S_orbital_med_olfact	0.39 (0.02, 0.77)	0.037	0.207
rh_G_insular_short	0.40 (0.02, 0.77)	0.036	0.207
rh_G_and_S_cingul_Mid_Post	0.39 (0.01, 0.76)	0.040	0.217
lh_G_cuneus	-0.36 (-0.73, 0.02)	0.058	0.243
lh_S_front_middle	0.36 (-0.02, 0.73)	0.058	0.243
lh_S_circular_insula_ant	0.36 (-0.02, 0.73)	0.058	0.243
rh_G_and_S_transv_frontopol	0.36 (-0.01, 0.74)	0.057	0.243
lh_S_oc_temp_med_and_Lingual	0.36 (-0.01, 0.74)	0.056	0.243
rh_G_and_S_subcentral	0.36 (-0.01, 0.74)	0.055	0.243
lh_G_and_S_frontomargin	0.37 (0.00, 0.74)	0.051	0.243
lh_S_temporal_inf	0.37 (0.00, 0.75)	0.049	0.243
lh_Pole_occipital	-0.35 (-0.72, 0.03)	0.066	0.249
rh_MeanThickness	0.35 (-0.03, 0.72)	0.066	0.249
lh_G_and_S_cingul_Mid_Ant	0.35 (-0.03, 0.72)	0.066	0.249
lh_G_front_sup	0.35 (-0.02, 0.72)	0.065	0.249
lh_MeanThickness	0.34 (-0.03, 0.71)	0.071	0.255

rh_G_cingul_Post_ventral	0.34 (-0.03, 0.72)	0.070	0.255
rh_G_pariet_inf_Supramar	0.34 (-0.04, 0.71)	0.075	0.262
rh_S_parieto_occipital	-0.33 (-0.70, 0.04)	0.081	0.269
lh_S_oc_temp_lat	0.33 (-0.05, 0.70)	0.082	0.269
lh_G_front_inf_Triangul	0.33 (-0.04, 0.71)	0.079	0.269
rh_G_oc_temp_lat_fusifor	0.33 (-0.05, 0.70)	0.084	0.270
rh_G_front_middle	0.32 (-0.06, 0.69)	0.095	0.279
lh_G_front_inf_Opercular	0.32 (-0.06, 0.69)	0.094	0.279
rh_G_precentral	0.32 (-0.06, 0.69)	0.092	0.279
lh_G_pariet_inf_Supramar	0.32 (-0.05, 0.69)	0.091	0.279
lh_G_and_S_paracentral	0.31 (-0.06, 0.68)	0.099	0.287
rh_S_suborbital	0.31 (-0.07, 0.68)	0.104	0.295
lh_Lat_Fis_post	0.29 (-0.08, 0.67)	0.120	0.333
lh_S_cingul_Marginalis	-0.27 (-0.65, 0.10)	0.149	0.362
lh_Lat_Fis_ant_Horizont	-0.27 (-0.64, 0.11)	0.158	0.362
lh_S_oc_middle_and_Lunatus	-0.26 (-0.63, 0.11)	0.166	0.362
lh_S_calcarine	-0.26 (-0.63, 0.11)	0.168	0.362
rh_S_collat_transv_post	-0.26 (-0.63, 0.11)	0.170	0.362
rh_S_oc_temp_lat	0.26 (-0.11, 0.63)	0.172	0.362
rh_S_circular_insula_ant	0.26 (-0.11, 0.63)	0.170	0.362
rh_S_circular_insula_inf	0.26 (-0.11, 0.63)	0.169	0.362
rh_G_temp_sup_G_T_transv	0.26 (-0.11, 0.64)	0.162	0.362
lh_S_circular_insula_inf	0.26 (-0.11, 0.64)	0.161	0.362
rh_G_orbital	0.27 (-0.11, 0.64)	0.156	0.362
lh_G_postcentral	0.27 (-0.10, 0.64)	0.155	0.362
rh_S_orbital_med_olfact	0.27 (-0.10, 0.64)	0.154	0.362
rh_G_front_inf_Triangul	0.27 (-0.10, 0.65)	0.147	0.362
rh_S_orbital_H_Shaped	0.28 (-0.09, 0.65)	0.141	0.362
lh_G_and_S_subcentral	0.28 (-0.09, 0.65)	0.138	0.362
rh_Lat_Fis_post	0.28 (-0.09, 0.65)	0.136	0.362
rh_G_subcallosal	-0.26 (-0.63, 0.12)	0.174	0.364
lh_G_oc_temp_lat_fusifor	0.25 (-0.12, 0.63)	0.178	0.367
lh_Lat_Fis_ant_Vertical	-0.25 (-0.62, 0.12)	0.184	0.373
rh_S_precentral_inf_part	0.24 (-0.13, 0.61)	0.207	0.388
lh_S_occipital_ant	0.24 (-0.13, 0.61)	0.206	0.388
lh_G_pariet_inf_Angular	0.24 (-0.13, 0.61)	0.205	0.388
rh_G_Ins_lg_and_S_cent_ins	0.24 (-0.13, 0.61)	0.202	0.388
lh_S_interm_prim_Jensen	0.24 (-0.13, 0.62)	0.198	0.388
rh_S_collat_transv_ant	0.24 (-0.13, 0.62)	0.195	0.388
lh_G_and_S_transv_frontopol	0.23 (-0.14, 0.61)	0.213	0.389
rh_S_front_sup	0.24 (-0.14, 0.61)	0.212	0.389
rh_S_oc_middle_and_Lunatus	-0.23 (-0.60, 0.15)	0.229	0.415
rh_G_temp_sup_Lateral	0.22 (-0.15, 0.60)	0.235	0.415
lh_S_circular_insula_sup	0.23 (-0.15, 0.60)	0.232	0.415

lh_S_intrapariet_and_P_trans	-0.22 (-0.59, 0.15)	0.238	0.415
rh_S_cingul_Marginalis	-0.22 (-0.59, 0.15)	0.241	0.415
lh_G_subcallosal	0.22 (-0.15, 0.59)	0.247	0.417
lh_S_orbital_H_Shaped	0.22 (-0.15, 0.59)	0.246	0.417
lh_G_Ins_lg_and_S_cent_ins	0.22 (-0.16, 0.59)	0.250	0.417
rh_G_oc_temp_med_Lingual	0.21 (-0.16, 0.58)	0.260	0.424
lh_S_temporal_transverse	0.21 (-0.16, 0.58)	0.259	0.424
lh_G_rectus	-0.21 (-0.58, 0.17)	0.273	0.432
lh_S_precentral_inf_part	0.21 (-0.17, 0.58)	0.273	0.432
rh_G_and_S_frontomargin	0.21 (-0.16, 0.58)	0.271	0.432
rh_G_front_inf_Opercular	0.20 (-0.17, 0.57)	0.287	0.439
rh_G_temporal_middle	0.20 (-0.17, 0.57)	0.286	0.439
rh_G_front_inf_Orbital	0.20 (-0.17, 0.57)	0.286	0.439
lh_S_collat_transv_ant	0.20 (-0.17, 0.57)	0.290	0.440
lh_S_parieto_occipital	-0.18 (-0.56, 0.19)	0.326	0.489
lh_G_occipital_sup	-0.18 (-0.56, 0.19)	0.330	0.490
rh_S_precentral_sup_part	0.17 (-0.20, 0.55)	0.354	0.520
rh_G_and_S_occipital_inf	0.16 (-0.21, 0.54)	0.382	0.550
rh_G_and_S_paracentral	0.17 (-0.21, 0.54)	0.379	0.550
lh_G_front_inf_Orbital	0.16 (-0.22, 0.53)	0.407	0.581
lh_G_temp_sup_Plan_tempo	0.15 (-0.22, 0.52)	0.416	0.582
rh_S_temporal_sup	0.15 (-0.22, 0.53)	0.412	0.582
rh_S_front_inf	0.15 (-0.22, 0.52)	0.420	0.583
rh_S_subparietal	-0.14 (-0.51, 0.23)	0.455	0.626
lh_G_and_S_occipital_inf	0.14 (-0.23, 0.51)	0.464	0.632
lh_S_precentral_sup_part	0.14 (-0.24, 0.51)	0.468	0.633
rh_S_orbital_lateral	0.13 (-0.24, 0.50)	0.480	0.642
rh_G_occipital_sup	-0.13 (-0.50, 0.24)	0.489	0.643
lh_S_temporal_sup	0.13 (-0.24, 0.50)	0.485	0.643
rh_Pole_occipital	-0.12 (-0.49, 0.25)	0.513	0.663
rh_S_interm_prim_Jensen	0.12 (-0.25, 0.49)	0.512	0.663
rh_S_circular_insula_sup	0.12 (-0.25, 0.49)	0.521	0.667
lh_S_front_sup	0.12 (-0.25, 0.49)	0.527	0.670
rh_S_oc_sup_and_transversal	-0.12 (-0.49, 0.25)	0.535	0.674
lh_G_and_S_cingul_Mid_Post	0.11 (-0.26, 0.48)	0.562	0.697
rh_S_occipital_ant	0.11 (-0.26, 0.48)	0.561	0.697
rh_S_calcarine	-0.10 (-0.47, 0.27)	0.585	0.719
rh_S_central	-0.09 (-0.47, 0.28)	0.614	0.749
lh_G_oc_temp_med_Lingual	-0.09 (-0.46, 0.29)	0.647	0.783
rh_S_intrapariet_and_P_trans	-0.08 (-0.45, 0.29)	0.657	0.783
rh_G_occipital_middle	0.08 (-0.29, 0.45)	0.658	0.783
lh_S_subparietal	0.08 (-0.29, 0.45)	0.670	0.791
lh_S_suborbital	-0.08 (-0.45, 0.29)	0.683	0.796
lh_S_collat_transv_post	-0.07 (-0.44, 0.30)	0.695	0.796

lh_G_parietal_sup	0.07 (-0.30, 0.45)	0.692	0.796
rh_G_precuneus	0.08 (-0.30, 0.45)	0.687	0.796
lh_S_oc_sup_and_transversal	-0.07 (-0.44, 0.30)	0.709	0.798
rh_S_temporal_inf	0.07 (-0.30, 0.44)	0.718	0.798
rh_Lat_Fis_ant_Horizont	0.07 (-0.30, 0.44)	0.717	0.798
lh_S_front_inf	0.07 (-0.30, 0.44)	0.715	0.798
lh_G_orbital	0.07 (-0.31, 0.44)	0.724	0.799
rh_G_pariet_inf_Angular	0.06 (-0.31, 0.43)	0.758	0.830
rh_S_temporal_transverse	0.06 (-0.32, 0.43)	0.768	0.835
rh_G_postcentral	0.05 (-0.32, 0.42)	0.790	0.853
rh_G_cuneus	-0.04 (-0.41, 0.33)	0.823	0.878
lh_S_central	0.04 (-0.33, 0.41)	0.825	0.878
lh_G_temporal_inf	0.02 (-0.35, 0.39)	0.902	0.953
lh_G_precuneus	-0.02 (-0.39, 0.35)	0.916	0.961
rh_G_temporal_inf	-0.02 (-0.39, 0.35)	0.927	0.966
lh_G_occipital_middle	0.01 (-0.36, 0.39)	0.939	0.971
rh_S_postcentral	0.01 (-0.36, 0.38)	0.960	0.986
rh_S_front_middle	0.00 (-0.37, 0.37)	0.992	0.993
lh_S_postcentral	0.00 (-0.37, 0.37)	0.992	0.993
rh_G_parietal_sup	0.00 (-0.37, 0.37)	0.993	0.993
rh_G_rectus	0.00 (-0.37, 0.38)	0.984	0.993

Table S2. Group differences in subcortical volumes between patients after COVID-19 and healthy control participants.

Region	Cohen's d (95% CI)	p	p (FDR)
Left Caudate	0.24 (-0.14, 0.61)	0.209	0.504
Right Caudate	0.17 (-0.20, 0.54)	0.364	0.504
Left Pallidum	0.46 (0.08, 0.83)	0.017	0.166
Right Pallidum	0.21 (-0.16, 0.58)	0.266	0.504
Left Putamen	0.06 (-0.31, 0.43)	0.749	0.749
Right Putamen	0.10 (-0.27, 0.47)	0.596	0.662
Left Thalamus	0.16 (-0.21, 0.53)	0.403	0.504
Right Thalamus	0.32 (-0.05, 0.70)	0.087	0.433

Table S3. Associations between cortical thickness and cognitive performance in patients after COVID-19, adjusted for sex, age, and years of education. *Abbreviations: lh - left hemisphere, rh - right hemisphere, S - sulcus, G - gyrus*.

Region	Coefficient (95% CI)	Std. coefficient ß (95% CI)	p
lh_S_orbital_med_olfact	5.73 (-0.60, 12.06)	0.29 (-0.03, 0.61)	0.085
lh_G_oc_temp_med_Parahip	-2.05 (-4.34, 0.24)	-0.26 (-0.55, 0.03)	0.089
lh_G_front_middle	6.86 (-0.84, 14.55)	0.31 (-0.04, 0.65)	0.090
rh_Pole_temporal	3.69 (-0.49, 7.87)	0.30 (-0.04, 0.64)	0.093
rh_G_temp_sup_Plan_tempo	3.71 (-0.54, 7.97)	0.30 (-0.04, 0.65)	0.096
rh_Lat_Fis_ant_Vertical	3.29 (-0.68, 7.27)	0.26 (-0.05, 0.57)	0.114
lh_G_temporal_middle	3.77 (-1.05, 8.60)	0.26 (-0.07, 0.60)	0.135
lh_Pole_temporal	3.22 (-0.90, 7.35)	0.23 (-0.06, 0.52)	0.135
lh_G_and_S_cingul_Ant	6.12 (-1.72, 13.97)	0.26 (-0.07, 0.60)	0.136
lh_S_orbital_lateral	3.54 (-1.08, 8.16)	0.27 (-0.08, 0.61)	0.142
lh_S_temporal_inf	4.25 (-1.55, 10.06)	0.24 (-0.09, 0.57)	0.160
rh_G_temp_sup_Plan_polar	2.69 (-1.49, 6.86)	0.28 (-0.16, 0.71)	0.216
rh_S_pericallosal	3.21 (-2.62, 9.05)	0.20 (-0.16, 0.57)	0.288
lh_S_pericallosal	2.45 (-2.65, 7.55)	0.15 (-0.17, 0.48)	0.354
lh_G_cingul_Post_dorsal	-2.00 (-6.19, 2.20)	-0.15 (-0.46, 0.16)	0.358
lh_G_temp_sup_G_T_transv	1.85 (-2.16, 5.86)	0.16 (-0.18, 0.50)	0.373
rh_G_oc_temp_med_Parahip	-1.22 (-4.04, 1.61)	-0.14 (-0.46, 0.18)	0.404
lh_G_temp_sup_Plan_polar	1.60 (-2.24, 5.45)	0.14 (-0.19, 0.47)	0.419
lh_G_cingul_Post_ventral	1.19 (-1.74, 4.12)	0.13 (-0.19, 0.46)	0.431
rh_G_insular_short	-2.08 (-7.61, 3.45)	-0.14 (-0.53, 0.24)	0.467
rh_G_front_sup	-2.58 (-10.13, 4.98)	-0.11 (-0.45, 0.22)	0.508
lh_G_temp_sup_Lateral	1.88 (-4.16, 7.92)	0.12 (-0.27, 0.52)	0.546
lh_G_insular_short	-0.80 (-4.72, 3.12)	-0.06 (-0.37, 0.24)	0.692
lh_G_precentral	-0.81 (-4.98, 3.36)	-0.07 (-0.41, 0.28)	0.705
rh_G_and_S_cingul_Ant	1.15 (-6.51, 8.81)	0.05 (-0.29, 0.39)	0.771
rh_G_cingul_Post_dorsal	-0.53 (-4.99, 3.93)	-0.04 (-0.38, 0.30)	0.818
rh_G_and_S_cingul_Mid_Ant	-0.78 (-7.64, 6.09)	-0.04 (-0.40, 0.31)	0.826
rh_G_and_S_cingul_Mid_Post	-0.40 (-8.34, 7.54)	-0.02 (-0.37, 0.34)	0.921
rh_S_oc_temp_med_and_Lingual	-0.30 (-7.20, 6.60)	-0.02 (-0.38, 0.35)	0.932

Table S3a. Associations between cortical thickness and cognitive performance in patients after COVID-19, adjusted for sex, age, years of education and scanner type. *Abbreviations: lh - left hemisphere, rh - right hemisphere, S - sulcus, G - gyrus*.

Region	Coefficient (95% CI)	Std. coefficient ß (95% CI)	p
lh_S_orbital_med_olfact	5.73 (-0.60, 12.06)	0.29 (-0.03, 0.61)	0.085
lh_G_oc_temp_med_Parahip	-2.05 (-4.34, 0.24)	-0.28 (-0.59, 0.03)	0.089
lh_G_front_middle	6.86 (-0.84, 14.55)	0.28 (-0.04, 0.60)	0.090
rh_Pole_temporal	3.69 (-0.49, 7.87)	0.28 (-0.04, 0.59)	0.093
rh_G_temp_sup_Plan_tempo	3.71 (-0.54, 7.97)	0.29 (-0.04, 0.62)	0.096
rh_Lat_Fis_ant_Vertical	3.29 (-0.68, 7.27)	0.27 (-0.06, 0.59)	0.114
lh_G_temporal_middle	3.77 (-1.05, 8.60)	0.26 (-0.07, 0.59)	0.135
lh_Pole_temporal	3.22 (-0.90, 7.35)	0.25 (-0.07, 0.57)	0.135
lh_G_and_S_cingul_Ant	6.12 (-1.72, 13.97)	0.27 (-0.07, 0.61)	0.136
lh_S_orbital_lateral	3.54 (-1.08, 8.16)	0.25 (-0.07, 0.57)	0.142
lh_S_temporal_inf	4.25 (-1.55, 10.06)	0.25 (-0.09, 0.59)	0.160
rh_G_temp_sup_Plan_polar	2.69 (-1.49, 6.86)	0.22 (-0.12, 0.57)	0.216
rh_S_pericallosal	3.21 (-2.62, 9.05)	0.19 (-0.15, 0.53)	0.288
lh_S_pericallosal	2.45 (-2.65, 7.55)	0.16 (-0.17, 0.48)	0.354
lh_G_cingul_Post_dorsal	-2.00 (-6.19, 2.20)	-0.16 (-0.49, 0.17)	0.358
lh_G_temp_sup_G_T_transv	1.85 (-2.16, 5.86)	0.15 (-0.18, 0.48)	0.373
rh_G_oc_temp_med_Parahip	-1.22 (-4.04, 1.61)	-0.14 (-0.47, 0.19)	0.404
lh_G_temp_sup_Plan_polar	1.60 (-2.24, 5.45)	0.15 (-0.20, 0.49)	0.419
lh_G_cingul_Post_ventral	1.19 (-1.74, 4.12)	0.14 (-0.20, 0.48)	0.431
rh_G_insular_short	-2.08 (-7.61, 3.45)	-0.12 (-0.46, 0.21)	0.467
rh_G_front_sup	-2.58 (-10.13, 4.98)	-0.12 (-0.46, 0.23)	0.508
lh_G_temp_sup_Lateral	1.88 (-4.16, 7.92)	0.11 (-0.25, 0.47)	0.546
lh_G_insular_short	-0.80 (-4.72, 3.12)	-0.07 (-0.40, 0.26)	0.692
lh_G_precentral	-0.81 (-4.98, 3.36)	-0.07 (-0.40, 0.27)	0.705
rh_G_and_S_cingul_Ant	1.15 (-6.51, 8.81)	0.05 (-0.28, 0.38)	0.771
rh_G_cingul_Post_dorsal	-0.53 (-4.99, 3.93)	-0.04 (-0.39, 0.31)	0.818
rh_G_and_S_cingul_Mid_Ant	-0.78 (-7.64, 6.09)	-0.04 (-0.41, 0.33)	0.826
rh_G_and_S_cingul_Mid_Post	-0.40 (-8.34, 7.54)	-0.02 (-0.36, 0.32)	0.921
rh_S_oc_temp_med_and_Lingual	-0.30 (-7.20, 6.60)	-0.02 (-0.39, 0.36)	0.932

Table S3b. Associations between cortical thickness and cognitive performance in patients after COVID-19, adjusted for sex, age, years of education and study site. *Abbreviations: lh - left hemisphere, rh - right hemisphere, S - sulcus, G - gyrus*.

Region	Coefficient (95% CI)	Std. coefficient ß (95% CI)	p
lh_G_oc_temp_med_Parahip	-3.14 (-5.75, -0.52)	-0.43 (-0.79, -0.07)	0.025
lh_S_orbital_med_olfact	5.90 (-0.52, 12.33)	0.30 (-0.03, 0.62)	0.081
rh_Lat_Fis_ant_Vertical	3.80 (-0.34, 7.94)	0.31 (-0.03, 0.65)	0.081
lh_G_front_middle	7.09 (-0.73, 14.90)	0.29 (-0.03, 0.62)	0.085
rh_G_temp_sup_Plan_tempo	3.70 (-0.61, 8.01)	0.29 (-0.05, 0.62)	0.102
rh_Pole_temporal	3.66 (-0.67, 7.99)	0.27 (-0.05, 0.60)	0.108
lh_G_and_S_cingul_Ant	6.61 (-1.42, 14.64)	0.29 (-0.06, 0.63)	0.116
lh_S_orbital_lateral	3.59 (-1.09, 8.27)	0.25 (-0.08, 0.57)	0.143
lh_S_temporal_inf	4.45 (-1.45, 10.36)	0.26 (-0.08, 0.61)	0.149
lh_G_temporal_middle	3.71 (-1.21, 8.63)	0.26 (-0.08, 0.59)	0.149
lh_Pole_temporal	3.28 (-1.14, 7.70)	0.25 (-0.09, 0.59)	0.156
rh_G_oc_temp_med_Parahip	-2.25 (-5.65, 1.15)	-0.26 (-0.65, 0.13)	0.204
rh_G_temp_sup_Plan_polar	2.62 (-1.72, 6.96)	0.22 (-0.14, 0.58)	0.245
lh_G_cingul_Post_dorsal	-2.61 (-7.11, 1.89)	-0.21 (-0.56, 0.15)	0.264
rh_S_pericallosal	3.21 (-2.71, 9.12)	0.19 (-0.16, 0.53)	0.296
lh_S_pericallosal	2.34 (-3.07, 7.76)	0.15 (-0.20, 0.50)	0.403
lh_G_temp_sup_G_T_transv	1.80 (-2.61, 6.20)	0.15 (-0.21, 0.51)	0.430
rh_G_insular_short	-2.22 (-7.84, 3.41)	-0.13 (-0.47, 0.20)	0.445
lh_G_temp_sup_Plan_polar	1.53 (-2.70, 5.75)	0.14 (-0.24, 0.52)	0.484
rh_G_front_sup	-2.75 (-10.43, 4.93)	-0.12 (-0.47, 0.22)	0.488
lh_G_cingul_Post_ventral	1.13 (-2.11, 4.38)	0.13 (-0.25, 0.51)	0.499
lh_G_temp_sup_Lateral	1.88 (-4.24, 7.99)	0.11 (-0.25, 0.48)	0.551
lh_G_insular_short	-1.24 (-5.49, 3.00)	-0.10 (-0.46, 0.25)	0.570
rh_G_cingul_Post_dorsal	-1.34 (-6.57, 3.90)	-0.11 (-0.52, 0.31)	0.620
lh_G_precentral	-0.97 (-5.24, 3.31)	-0.08 (-0.42, 0.27)	0.660
rh_G_and_S_cingul_Ant	1.70 (-6.35, 9.75)	0.07 (-0.27, 0.42)	0.682
rh_G_and_S_cingul_Mid_Ant	-0.80 (-7.76, 6.15)	-0.04 (-0.42, 0.33)	0.822
rh_S_oc_temp_med_and_Lingual	-0.64 (-7.81, 6.52)	-0.04 (-0.43, 0.36)	0.861
rh_G_and_S_cingul_Mid_Post	-0.04 (-8.30, 8.23)	-0.00 (-0.36, 0.35)	0.993

Table S3c. Associations between cortical thickness and cognitive performance in patients after COVID-19, adjusted for sex, age, years of education and comorbidity. *Abbreviations: lh - left hemisphere, rh - right hemisphere, S - sulcus, G - gyrus*.

Region	Coefficient (95% CI)	Std. coefficient ß (95% CI)	p
lh_G_oc_temp_med_Parahip	-2.52 (-4.81, -0.23)	-0.32 (-0.61, -0.03)	0.038
lh_S_orbital_lateral	3.98 (-0.61, 8.57)	0.30 (-0.05, 0.64)	0.099
lh_G_front_middle	6.55 (-1.15, 14.24)	0.29 (-0.05, 0.64)	0.105
rh_Pole_temporal	3.48 (-0.71, 7.67)	0.28 (-0.06, 0.63)	0.113
lh_S_orbital_med_olfact	5.26 (-1.15, 11.67)	0.27 (-0.06, 0.59)	0.117
rh_G_temp_sup_Plan_tempo	3.48 (-0.79, 7.75)	0.28 (-0.06, 0.63)	0.120
rh_Lat_Fis_ant_Vertical	3.10 (-0.89, 7.08)	0.24 (-0.07, 0.56)	0.137
lh_G_cingul_Post_dorsal	-3.31 (-7.68, 1.07)	-0.25 (-0.57, 0.08)	0.149
rh_G_temp_sup_Plan_polar	3.08 (-1.08, 7.23)	0.32 (-0.11, 0.75)	0.157
lh_S_temporal_inf	4.07 (-1.72, 9.86)	0.23 (-0.10, 0.56)	0.178
lh_G_and_S_cingul_Ant	5.41 (-2.59, 13.42)	0.23 (-0.11, 0.58)	0.195
lh_Pole_temporal	2.81 (-1.43, 7.05)	0.20 (-0.10, 0.50)	0.203
lh_G_temporal_middle	3.14 (-2.02, 8.29)	0.22 (-0.14, 0.58)	0.242
rh_S_pericallosal	3.17 (-2.64, 8.97)	0.20 (-0.17, 0.56)	0.293
rh_G_oc_temp_med_Parahip	-1.54 (-4.37, 1.29)	-0.17 (-0.50, 0.15)	0.295
rh_G_cingul_Post_dorsal	-2.15 (-7.06, 2.77)	-0.16 (-0.54, 0.21)	0.398
rh_G_front_sup	-3.03 (-10.56, 4.49)	-0.14 (-0.47, 0.20)	0.435
rh_G_insular_short	-2.07 (-7.57, 3.43)	-0.14 (-0.52, 0.24)	0.466
lh_G_insular_short	-1.49 (-5.51, 2.52)	-0.12 (-0.43, 0.20)	0.471
lh_G_temp_sup_Plan_polar	1.40 (-2.44, 5.25)	0.12 (-0.21, 0.46)	0.479
lh_S_pericallosal	1.70 (-3.65, 7.04)	0.11 (-0.23, 0.44)	0.538
lh_G_precentral	-1.17 (-5.35, 3.00)	-0.10 (-0.44, 0.25)	0.585
lh_G_cingul_Post_ventral	0.82 (-2.19, 3.83)	0.09 (-0.24, 0.43)	0.596
lh_G_temp_sup_Lateral	1.56 (-4.48, 7.60)	0.10 (-0.29, 0.50)	0.616
lh_G_temp_sup_G_T_transv	1.07 (-3.30, 5.45)	0.09 (-0.28, 0.47)	0.634
rh_G_and_S_cingul_Mid_Post	-1.37 (-9.41, 6.67)	-0.06 (-0.42, 0.30)	0.740
rh_S_oc_temp_med_and_Lingual	-0.92 (-7.84, 6.01)	-0.05 (-0.42, 0.32)	0.797
rh_G_and_S_cingul_Ant	0.90 (-6.73, 8.53)	0.04 (-0.30, 0.38)	0.819
rh_G_and_S_cingul_Mid_Ant	0.04 (-6.94, 7.01)	0.00 (-0.36, 0.36)	0.992

Table S3d. Associations between cortical thickness and cognitive performance in patients after COVID-19, adjusted for sex, age, years of education and body mass index. *Abbreviations: lh - left hemisphere, rh - right hemisphere, S - sulcus, G - gyrus*.

Region	Coefficient (95% CI)	Std. coefficient ß (95% CI)	p
lh_S_orbital_med_olfact	7.66 (0.51, 14.81)	0.39 (0.03, 0.75)	0.045
rh_Pole_temporal	3.84 (-1.03, 8.72)	0.31 (-0.08, 0.71)	0.134
lh_G_oc_temp_med_Parahip	-1.92 (-4.40, 0.57)	-0.24 (-0.56, 0.07)	0.142
lh_G_front_middle	6.38 (-2.15, 14.92)	0.29 (-0.10, 0.67)	0.154
rh_G_temp_sup_Plan_tempo	3.91 (-1.44, 9.25)	0.32 (-0.12, 0.75)	0.163
lh_G_temporal_middle	3.79 (-1.93, 9.51)	0.26 (-0.13, 0.66)	0.205
rh_G_insular_short	-3.88 (-10.25, 2.49)	-0.27 (-0.71, 0.17)	0.243
lh_S_orbital_lateral	3.14 (-2.06, 8.34)	0.24 (-0.15, 0.63)	0.247
lh_G_cingul_Post_dorsal	-2.53 (-7.13, 2.08)	-0.19 (-0.53, 0.15)	0.291
lh_G_insular_short	-2.30 (-6.58, 1.98)	-0.18 (-0.51, 0.15)	0.301
lh_S_temporal_inf	3.65 (-3.20, 10.51)	0.21 (-0.18, 0.60)	0.305
lh_Pole_temporal	2.70 (-2.56, 7.96)	0.19 (-0.18, 0.56)	0.323
rh_Lat_Fis_ant_Vertical	2.31 (-2.64, 7.26)	0.18 (-0.21, 0.57)	0.368
rh_G_front_sup	-3.78 (-11.97, 4.41)	-0.17 (-0.53, 0.20)	0.373
rh_G_and_S_cingul_Mid_Post	-4.35 (-14.04, 5.34)	-0.20 (-0.63, 0.24)	0.386
rh_G_oc_temp_med_Parahip	-1.24 (-4.30, 1.82)	-0.14 (-0.49, 0.21)	0.434
rh_G_temp_sup_Plan_polar	1.71 (-3.07, 6.50)	0.18 (-0.32, 0.68)	0.489
lh_G_and_S_cingul_Ant	3.56 (-7.53, 14.65)	0.15 (-0.32, 0.63)	0.534
lh_G_precentral	-1.44 (-6.23, 3.36)	-0.12 (-0.51, 0.28)	0.561
lh_G_temp_sup_G_T_transv	1.33 (-4.15, 6.81)	0.11 (-0.35, 0.58)	0.638
lh_G_cingul_Post_ventral	0.78 (-2.88, 4.45)	0.09 (-0.32, 0.50)	0.678
rh_G_and_S_cingul_Ant	-1.68 (-10.94, 7.58)	-0.07 (-0.49, 0.34)	0.725
lh_S_pericallosal	1.11 (-5.64, 7.85)	0.07 (-0.36, 0.50)	0.750
lh_G_temp_sup_Plan_polar	0.69 (-3.64, 5.02)	0.06 (-0.32, 0.44)	0.757
rh_S_pericallosal	0.92 (-6.57, 8.42)	0.06 (-0.41, 0.53)	0.811
rh_S_oc_temp_med_and_Lingual	-0.77 (-9.09, 7.55)	-0.04 (-0.48, 0.40)	0.857
rh_G_cingul_Post_dorsal	-0.43 (-5.47, 4.61)	-0.03 (-0.42, 0.35)	0.868
lh_G_temp_sup_Lateral	0.35 (-6.80, 7.50)	0.02 (-0.44, 0.49)	0.924
rh_G_and_S_cingul_Mid_Ant	-0.28 (-7.97, 7.41)	-0.01 (-0.41, 0.38)	0.944

Table S4a. Associations between cortical thickness and fatigue severity in patients after COVID-19, adjusted for sex, age, and scanner type. *Abbreviations: lh - left hemisphere, rh - right hemisphere, S - sulcus, G - gyrus*.

Region	Coefficient (95% CI)	Std. coefficient ß (95% CI)	p
lh_G_and_S_cingul_Ant	-76.07 (-113.44, -38.71)	-0.60 (-0.90, -0.31)	< 0.001
lh_Pole_temporal	-47.89 (-75.88, -19.90)	-0.63 (-0.99, -0.26)	0.002
lh_G_temp_sup_G_T_transv	-24.87 (-43.29, -6.45)	-0.39 (-0.68, -0.10)	0.012
rh_G_cingul_Post_dorsal	-33.28 (-59.72, -6.84)	-0.47 (-0.84, -0.10)	0.019
rh_S_pericallosal	-35.89 (-68.21, -3.57)	-0.42 (-0.79, -0.04)	0.036
lh_G_cingul_Post_ventral	-16.11 (-31.01, -1.22)	-0.33 (-0.64, -0.02)	0.041
rh_G_and_S_cingul_Mid_Post	-41.78 (-83.12, -0.45)	-0.35 (-0.69, -0.00)	0.055
lh_G_cingul_Post_dorsal	-21.50 (-46.10, 3.11)	-0.29 (-0.63, 0.04)	0.095
rh_G_and_S_cingul_Ant	-28.19 (-69.00, 12.63)	-0.23 (-0.57, 0.10)	0.184
lh_S_orbital_lateral	15.93 (-8.59, 40.46)	0.22 (-0.12, 0.56)	0.211
rh_G_front_sup	-27.95 (-73.56, 17.66)	-0.23 (-0.61, 0.15)	0.238
lh_G_temp_sup_Lateral	-16.57 (-44.52, 11.37)	-0.20 (-0.54, 0.14)	0.253
rh_G_insular_short	12.75 (-13.12, 38.63)	0.16 (-0.17, 0.49)	0.340
rh_Pole_temporal	-12.36 (-38.29, 13.58)	-0.19 (-0.58, 0.20)	0.357
lh_S_pericallosal	-9.48 (-35.39, 16.42)	-0.11 (-0.41, 0.19)	0.478
lh_G_front_middle	16.47 (-29.25, 62.18)	0.14 (-0.24, 0.51)	0.485
rh_G_temp_sup_Plan_tempo	-9.60 (-36.47, 17.26)	-0.14 (-0.55, 0.26)	0.488
rh_S_oc_temp_med_and_Lingual	-13.40 (-52.35, 25.55)	-0.13 (-0.51, 0.25)	0.504
lh_S_temporal_inf	11.14 (-21.83, 44.11)	0.12 (-0.23, 0.46)	0.512
rh_G_and_S_cingul_Mid_Ant	-15.22 (-60.99, 30.56)	-0.15 (-0.58, 0.29)	0.519
rh_G_oc_temp_med_Parahip	-5.39 (-24.30, 13.51)	-0.11 (-0.51, 0.28)	0.579
lh_G_precentral	5.73 (-16.61, 28.06)	0.09 (-0.25, 0.42)	0.618
lh_G_temp_sup_Plan_polar	-4.61 (-25.67, 16.45)	-0.07 (-0.41, 0.26)	0.671
lh_S_orbital_med_olfact	-6.35 (-43.75, 31.05)	-0.06 (-0.41, 0.29)	0.741
rh_Lat_Fis_ant_Vertical	-3.58 (-26.93, 19.77)	-0.05 (-0.39, 0.29)	0.766
lh_G_temporal_middle	-3.96 (-35.00, 27.07)	-0.05 (-0.45, 0.35)	0.804
rh_G_temp_sup_Plan_polar	-2.05 (-20.44, 16.34)	-0.04 (-0.39, 0.31)	0.828
lh_G_insular_short	-1.00 (-22.58, 20.58)	-0.01 (-0.33, 0.30)	0.928
lh_G_oc_temp_med_Parahip	-0.59 (-15.68, 14.50)	-0.01 (-0.36, 0.34)	0.940

Table S4b. Associations between cortical thickness and fatigue severity in patients after COVID-19, adjusted for sex, age, and study site. *Abbreviations: lh - left hemisphere, rh - right hemisphere, S - sulcus, G - gyrus*.

Region	Coefficient (95% CI)	Std. coefficient ß (95% CI)	p
lh_G_and_S_cingul_Ant	-80.43 (-118.14, -42.72)	-0.64 (-0.94, -0.34)	< 0.001
lh_Pole_temporal	-50.10 (-79.63, -20.58)	-0.65 (-1.04, -0.27)	0.002
lh_G_temp_sup_G_T_transv	-27.48 (-47.68, -7.29)	-0.43 (-0.75, -0.11)	0.012
rh_G_cingul_Post_dorsal	-36.47 (-65.44, -7.50)	-0.51 (-0.92, -0.10)	0.019
rh_S_pericallosal	-36.82 (-69.55, -4.09)	-0.43 (-0.80, -0.05)	0.034
lh_G_cingul_Post_ventral	-16.45 (-32.15, -0.74)	-0.34 (-0.66, -0.02)	0.048
rh_G_and_S_cingul_Mid_Post	-44.04 (-86.11, -1.98)	-0.36 (-0.71, -0.02)	0.048
lh_G_cingul_Post_dorsal	-21.43 (-47.10, 4.24)	-0.29 (-0.65, 0.06)	0.111
rh_G_and_S_cingul_Ant	-31.12 (-73.06, 10.82)	-0.26 (-0.60, 0.09)	0.155
lh_S_orbital_lateral	15.60 (-9.34, 40.53)	0.22 (-0.13, 0.56)	0.228
rh_G_front_sup	-27.81 (-73.98, 18.35)	-0.23 (-0.61, 0.15)	0.246
lh_G_temp_sup_Lateral	-16.12 (-44.60, 12.36)	-0.19 (-0.54, 0.15)	0.275
rh_G_insular_short	13.30 (-12.95, 39.54)	0.17 (-0.17, 0.50)	0.328
rh_Pole_temporal	-11.83 (-39.59, 15.94)	-0.18 (-0.60, 0.24)	0.409
lh_G_front_middle	15.56 (-31.04, 62.17)	0.13 (-0.26, 0.51)	0.517
rh_G_temp_sup_Plan_tempo	-8.99 (-36.47, 18.50)	-0.13 (-0.55, 0.28)	0.526
rh_G_and_S_cingul_Mid_Ant	-15.05 (-61.38, 31.28)	-0.14 (-0.59, 0.30)	0.529
lh_S_temporal_inf	10.81 (-22.60, 44.23)	0.11 (-0.24, 0.46)	0.530
rh_S_oc_temp_med_and_Lingual	-12.61 (-52.33, 27.10)	-0.12 (-0.51, 0.27)	0.538
lh_S_pericallosal	-8.64 (-36.45, 19.17)	-0.10 (-0.42, 0.22)	0.547
lh_G_precentral	6.87 (-16.12, 29.87)	0.10 (-0.24, 0.45)	0.562
rh_Lat_Fis_ant_Vertical	-5.15 (-29.55, 19.25)	-0.07 (-0.43, 0.28)	0.682
rh_G_oc_temp_med_Parahip	-4.57 (-26.74, 17.61)	-0.10 (-0.56, 0.37)	0.689
lh_S_orbital_med_olfact	-5.94 (-43.85, 31.97)	-0.06 (-0.41, 0.30)	0.761
lh_G_temp_sup_Plan_polar	-3.31 (-27.14, 20.51)	-0.05 (-0.44, 0.33)	0.787
lh_G_temporal_middle	-3.23 (-34.88, 28.42)	-0.04 (-0.45, 0.36)	0.843
lh_G_oc_temp_med_Parahip	0.81 (-15.88, 17.50)	0.02 (-0.37, 0.41)	0.925
rh_G_temp_sup_Plan_polar	-0.92 (-20.58, 18.73)	-0.02 (-0.39, 0.36)	0.927
lh_G_insular_short	0.86 (-22.82, 24.54)	0.01 (-0.33, 0.35)	0.943

Table S4c. Associations between cortical thickness and fatigue severity in patients after COVID-19, adjusted for sex, age, and comorbidity. *Abbreviations: lh - left hemisphere, rh - right hemisphere, S - sulcus, G - gyrus*.

Region	Coefficient (95% CI)	Std. coefficient ß (95% CI)	p
lh_G_and_S_cingul_Ant	-62.76 (-102.45, -23.07)	-0.50 (-0.81, -0.18)	0.004
lh_Pole_temporal	-26.51 (-58.99, 5.96)	-0.35 (-0.77, 0.08)	0.118
rh_G_and_S_cingul_Mid_Post	-30.40 (-68.83, 8.02)	-0.25 (-0.57, 0.07)	0.130
lh_G_temp_sup_G_T_transv	-14.70 (-34.85, 5.45)	-0.23 (-0.55, 0.09)	0.161
rh_G_insular_short	14.54 (-7.64, 36.71)	0.19 (-0.10, 0.47)	0.207
rh_S_pericallosal	-21.55 (-54.76, 11.67)	-0.25 (-0.63, 0.14)	0.212
lh_G_insular_short	11.82 (-8.88, 32.51)	0.17 (-0.13, 0.47)	0.271
rh_G_cingul_Post_dorsal	-14.66 (-44.04, 14.73)	-0.21 (-0.62, 0.21)	0.335
rh_G_and_S_cingul_Ant	-17.46 (-52.50, 17.58)	-0.14 (-0.43, 0.14)	0.335
lh_S_temporal_inf	14.15 (-15.37, 43.67)	0.15 (-0.16, 0.46)	0.354
lh_G_cingul_Post_ventral	-7.06 (-23.50, 9.39)	-0.14 (-0.48, 0.19)	0.406
lh_G_front_middle	16.46 (-23.97, 56.90)	0.14 (-0.20, 0.47)	0.430
lh_S_orbital_lateral	9.01 (-14.07, 32.09)	0.12 (-0.20, 0.45)	0.449
lh_G_oc_temp_med_Parahip	5.24 (-8.27, 18.74)	0.12 (-0.19, 0.44)	0.452
rh_G_front_sup	-15.79 (-58.57, 27.00)	-0.13 (-0.48, 0.22)	0.474
lh_G_precentral	6.77 (-13.44, 26.98)	0.10 (-0.20, 0.41)	0.516
lh_G_temp_sup_Plan_polar	6.19 (-14.00, 26.38)	0.10 (-0.23, 0.42)	0.552
lh_S_orbital_med_olfact	-9.29 (-42.09, 23.51)	-0.09 (-0.39, 0.22)	0.582
lh_S_pericallosal	7.49 (-18.98, 33.96)	0.09 (-0.22, 0.40)	0.583
lh_G_cingul_Post_dorsal	-6.95 (-31.99, 18.08)	-0.10 (-0.44, 0.25)	0.589
rh_Pole_temporal	-5.20 (-29.45, 19.04)	-0.08 (-0.44, 0.29)	0.676
rh_G_and_S_cingul_Mid_Ant	-7.56 (-48.89, 33.76)	-0.07 (-0.47, 0.32)	0.722
rh_G_oc_temp_med_Parahip	2.91 (-14.81, 20.62)	0.06 (-0.31, 0.43)	0.750
rh_G_temp_sup_Plan_polar	2.70 (-14.29, 19.69)	0.05 (-0.27, 0.38)	0.757
lh_G_temp_sup_Lateral	-4.15 (-31.51, 23.22)	-0.05 (-0.38, 0.28)	0.768
lh_G_temporal_middle	3.48 (-25.13, 32.08)	0.04 (-0.32, 0.41)	0.813
rh_Lat_Fis_ant_Vertical	-2.38 (-23.51, 18.75)	-0.03 (-0.34, 0.27)	0.826
rh_G_temp_sup_Plan_tempo	-2.77 (-27.39, 21.85)	-0.04 (-0.41, 0.33)	0.827
rh_S_oc_temp_med_and_Lingual	2.19 (-35.11, 39.49)	0.02 (-0.35, 0.39)	0.909

Table S4d. Associations between cortical thickness and fatigue severity in patients after COVID-19, adjusted for sex, age, and body mass index. *Abbreviations: lh - left hemisphere, rh - right hemisphere, S - sulcus, G - gyrus*.

Region	Coefficient (95% CI)	Std. coefficient ß (95% CI)	p
lh_G_and_S_cingul_Ant	-65.12 (-121.89, -8.36)	-0.52 (-0.97, -0.07)	0.032
lh_G_temp_sup_G_T_transv	-23.70 (-47.53, 0.14)	-0.37 (-0.75, 0.00)	0.061
rh_G_cingul_Post_dorsal	-25.65 (-54.42, 3.13)	-0.36 (-0.76, 0.04)	0.091
lh_Pole_temporal	-30.69 (-65.58, 4.21)	-0.40 (-0.86, 0.05)	0.095
rh_G_insular_short	23.00 (-3.24, 49.23)	0.29 (-0.04, 0.63)	0.096
lh_S_orbital_lateral	16.89 (-8.44, 42.21)	0.23 (-0.12, 0.59)	0.201
lh_G_front_middle	27.11 (-17.68, 71.90)	0.22 (-0.15, 0.59)	0.245
lh_G_precentral	14.37 (-10.97, 39.71)	0.22 (-0.17, 0.60)	0.275
lh_G_cingul_Post_ventral	-9.47 (-27.22, 8.27)	-0.19 (-0.56, 0.17)	0.304
lh_G_cingul_Post_dorsal	-12.60 (-38.73, 13.53)	-0.17 (-0.53, 0.19)	0.352
rh_G_and_S_cingul_Mid_Ant	-24.49 (-76.03, 27.05)	-0.23 (-0.72, 0.26)	0.359
rh_S_oc_temp_med_and_Lingual	-19.18 (-61.05, 22.69)	-0.19 (-0.60, 0.22)	0.377
lh_G_insular_short	9.46 (-12.52, 31.44)	0.14 (-0.18, 0.45)	0.406
lh_S_orbital_med_olfact	-17.77 (-59.77, 24.23)	-0.17 (-0.56, 0.23)	0.414
lh_S_temporal_inf	14.81 (-20.33, 49.96)	0.16 (-0.21, 0.52)	0.415
rh_G_and_S_cingul_Mid_Post	-20.46 (-70.68, 29.76)	-0.17 (-0.59, 0.25)	0.431
lh_G_temp_sup_Plan_polar	8.97 (-14.14, 32.08)	0.14 (-0.23, 0.52)	0.453
rh_S_pericallosal	-15.74 (-57.99, 26.51)	-0.18 (-0.67, 0.31)	0.471
rh_G_front_sup	-21.28 (-79.29, 36.72)	-0.18 (-0.65, 0.30)	0.478
rh_Lat_Fis_ant_Vertical	8.65 (-18.11, 35.42)	0.13 (-0.26, 0.52)	0.531
rh_G_temp_sup_Plan_tempo	-8.79 (-39.23, 21.64)	-0.13 (-0.59, 0.32)	0.576
lh_S_pericallosal	6.44 (-23.60, 36.48)	0.07 (-0.27, 0.42)	0.678
rh_G_temp_sup_Plan_polar	3.41 (-17.56, 24.39)	0.07 (-0.34, 0.47)	0.752
lh_G_oc_temp_med_Parahip	1.89 (-12.72, 16.49)	0.04 (-0.30, 0.38)	0.802
rh_Pole_temporal	-3.31 (-38.46, 31.84)	-0.05 (-0.58, 0.48)	0.855
rh_G_oc_temp_med_Parahip	-1.34 (-20.21, 17.53)	-0.03 (-0.42, 0.37)	0.890
rh_G_and_S_cingul_Ant	-2.63 (-44.56, 39.29)	-0.02 (-0.37, 0.32)	0.903
lh_G_temp_sup_Lateral	-1.67 (-35.50, 32.17)	-0.02 (-0.43, 0.39)	0.924
lh_G_temporal_middle	0.48 (-40.72, 41.68)	0.01 (-0.52, 0.53)	0.982

Table S5a. Associations between cortical thickness and COVID-19 severity (WHO ordinal scale) in patients after COVID-19, adjusted for sex, age, and scanner type. *Abbreviations: lh - left hemisphere, rh - right hemisphere, S - sulcus, G - gyrus*.

Region	Coefficient (95% CI)	p
lh_S_orbital_med_olfact	-5.52 (-9.38, -1.65)	0.005
rh_Lat_Fis_ant_Vertical	-3.02 (-5.27, -0.77)	0.009
lh_G_oc_temp_med_Parahip	1.91 (0.46, 3.35)	0.010
lh_G_front_middle	-5.26 (-9.50, -1.01)	0.015
lh_G_temp_sup_Lateral	-3.26 (-5.98, -0.55)	0.018
rh_G_front_sup	-4.24 (-8.34, -0.14)	0.043
lh_G_temporal_middle	-2.65 (-5.30, -0.00)	0.050
rh_G_temp_sup_Plan_tempo	-2.10 (-4.22, 0.01)	0.051
rh_G_oc_temp_med_Parahip	1.43 (-0.15, 3.01)	0.076
lh_S_temporal_inf	-2.77 (-5.92, 0.39)	0.086
lh_G_cingul_Post_ventral	1.22 (-0.20, 2.64)	0.092
lh_G_and_S_cingul_Ant	-3.50 (-7.61, 0.61)	0.095
rh_G_and_S_cingul_Mid_Post	-3.24 (-7.11, 0.63)	0.101
rh_G_cingul_Post_dorsal	1.90 (-0.58, 4.38)	0.133
rh_Pole_temporal	-1.40 (-3.38, 0.58)	0.167
lh_S_orbital_lateral	-1.40 (-3.55, 0.76)	0.203
rh_G_and_S_cingul_Ant	-2.28 (-6.08, 1.53)	0.241
lh_G_insular_short	1.31 (-0.91, 3.52)	0.249
rh_G_temp_sup_Plan_polar	-0.84 (-2.35, 0.68)	0.279
rh_S_oc_temp_med_and_Lingual	-1.74 (-4.95, 1.47)	0.288
lh_Pole_temporal	-1.10 (-3.38, 1.18)	0.344
rh_G_and_S_cingul_Mid_Ant	-1.61 (-5.37, 2.16)	0.403
rh_S_pericallosal	-1.04 (-3.89, 1.81)	0.474
lh_G_cingul_Post_dorsal	0.54 (-1.76, 2.83)	0.646
lh_S_pericallosal	0.51 (-1.92, 2.94)	0.682
lh_G_temp_sup_Plan_polar	-0.35 (-2.22, 1.51)	0.711
rh_G_insular_short	-0.25 (-2.70, 2.20)	0.843
lh_G_temp_sup_G_T_transv	-0.16 (-2.14, 1.82)	0.871
lh_G_precentral	-0.00 (-2.23, 2.23)	0.999

Table S5b. Associations between cortical thickness and COVID-19 severity (WHO ordinal scale) in patients after COVID-19, adjusted for sex, age, and study site. *Abbreviations: lh - left hemisphere, rh - right hemisphere, S - sulcus, G - gyrus*.

Region	Coefficient (95% CI)	p
lh_S_orbital_med_olfact	-5.49 (-9.60, -1.38)	0.009
lh_G_temp_sup_Lateral	-3.60 (-6.48, -0.73)	0.014
lh_G_temp_sup_Plan_polar	-2.65 (-4.98, -0.32)	0.026
lh_Pole_temporal	-2.58 (-5.04, -0.12)	0.040
lh_G_front_middle	-4.75 (-9.32, -0.19)	0.041
rh_Lat_Fis_ant_Vertical	-2.38 (-4.67, -0.09)	0.042
lh_G_temporal_middle	-2.72 (-5.40, -0.05)	0.046
rh_G_front_sup	-4.27 (-8.46, -0.08)	0.046
rh_Pole_temporal	-2.09 (-4.23, 0.04)	0.055
rh_G_temp_sup_Plan_polar	-1.61 (-3.31, 0.09)	0.064
rh_G_temp_sup_Plan_tempo	-1.91 (-4.01, 0.19)	0.074
lh_S_temporal_inf	-2.90 (-6.18, 0.38)	0.083
rh_S_oc_temp_med_and_Lingual	-2.92 (-6.44, 0.60)	0.104
lh_G_and_S_cingul_Ant	-2.98 (-7.26, 1.30)	0.172
lh_S_orbital_lateral	-1.29 (-3.62, 1.04)	0.277
rh_G_and_S_cingul_Mid_Post	-2.17 (-6.14, 1.80)	0.285
lh_G_oc_temp_med_Parahip	0.84 (-0.82, 2.51)	0.321
rh_S_pericallosal	-1.28 (-4.24, 1.68)	0.397
rh_G_insular_short	-1.06 (-3.66, 1.54)	0.423
lh_G_temp_sup_G_T_transv	-0.74 (-2.75, 1.27)	0.471
lh_G_cingul_Post_ventral	0.47 (-1.08, 2.01)	0.553
rh_G_and_S_cingul_Mid_Ant	-1.04 (-4.72, 2.63)	0.579
rh_G_and_S_cingul_Ant	-1.01 (-4.98, 2.97)	0.619
lh_G_cingul_Post_dorsal	-0.61 (-3.10, 1.88)	0.630
lh_G_precentral	-0.56 (-2.87, 1.75)	0.634
lh_S_pericallosal	-0.60 (-3.13, 1.94)	0.645
lh_G_insular_short	-0.35 (-2.82, 2.12)	0.782
rh_G_cingul_Post_dorsal	0.20 (-2.60, 2.99)	0.890
rh_G_oc_temp_med_Parahip	0.03 (-1.84, 1.91)	0.972

Table S5c. Associations between cortical thickness and COVID-19 severity (WHO ordinal scale) in patients after COVID-19, adjusted for sex, age, and comorbidity. *Abbreviations: lh - left hemisphere, rh - right hemisphere, S - sulcus, G - gyrus*.

Region	Coefficient (95% CI)	p
lh_G_temp_sup_Lateral	-4.20 (-7.09, -1.31)	0.004
rh_Lat_Fis_ant_Vertical	-3.43 (-5.83, -1.04)	0.005
lh_S_orbital_med_olfact	-5.32 (-9.17, -1.48)	0.007
lh_G_front_middle	-5.36 (-9.59, -1.13)	0.013
lh_G_oc_temp_med_Parahip	1.61 (0.21, 3.01)	0.024
rh_G_temp_sup_Plan_tempo	-2.46 (-4.61, -0.31)	0.025
lh_G_temporal_middle	-3.04 (-5.75, -0.33)	0.028
rh_G_front_sup	-4.45 (-8.63, -0.27)	0.037
lh_G_and_S_cingul_Ant	-4.26 (-8.55, 0.03)	0.051
lh_S_temporal_inf	-2.76 (-5.85, 0.33)	0.080
rh_G_oc_temp_med_Parahip	1.38 (-0.18, 2.93)	0.083
rh_G_and_S_cingul_Mid_Post	-3.38 (-7.24, 0.48)	0.086
lh_Pole_temporal	-1.70 (-4.06, 0.67)	0.159
rh_S_oc_temp_med_and_Lingual	-2.33 (-5.70, 1.03)	0.174
rh_Pole_temporal	-1.31 (-3.31, 0.69)	0.199
lh_G_cingul_Post_ventral	0.97 (-0.52, 2.45)	0.202
lh_S_orbital_lateral	-1.34 (-3.49, 0.82)	0.225
rh_G_temp_sup_Plan_polar	-0.90 (-2.47, 0.67)	0.261
rh_G_and_S_cingul_Ant	-1.97 (-5.61, 1.68)	0.290
rh_G_cingul_Post_dorsal	1.22 (-1.34, 3.77)	0.350
rh_S_pericallosal	-1.36 (-4.22, 1.50)	0.352
lh_G_temp_sup_G_T_transv	-1.06 (-3.31, 1.18)	0.353
lh_G_insular_short	0.93 (-1.39, 3.25)	0.431
rh_G_and_S_cingul_Mid_Ant	-1.31 (-4.97, 2.35)	0.484
lh_G_temp_sup_Plan_polar	-0.59 (-2.51, 1.33)	0.546
rh_G_insular_short	-0.15 (-2.53, 2.24)	0.904
lh_G_precentral	0.13 (-2.07, 2.33)	0.910
lh_G_cingul_Post_dorsal	0.12 (-2.16, 2.39)	0.920
lh_S_pericallosal	-0.12 (-2.68, 2.44)	0.928

Table S5d. Associations between cortical thickness and COVID-19 severity (WHO ordinal scale) in patients after COVID-19, adjusted for sex, age, and body mass index. *Abbreviations: lh - left hemisphere, rh - right hemisphere, S - sulcus, G - gyrus*.

Region	Coefficient (95% CI)	p
rh_Lat_Fis_ant_Vertical	-3.10 (-5.69, -0.51)	0.019
lh_G_and_S_cingul_Ant	-6.08 (-11.35, -0.82)	0.023
lh_G_insular_short	2.79 (0.31, 5.27)	0.027
lh_G_oc_temp_med_Parahip	1.50 (0.02, 2.99)	0.048
lh_G_front_middle	-4.59 (-9.16, -0.02)	0.049
lh_G_precentral	2.16 (-0.40, 4.72)	0.098
lh_S_orbital_lateral	-1.83 (-4.29, 0.64)	0.146
lh_S_orbital_med_olfact	-2.92 (-7.13, 1.30)	0.175
rh_G_and_S_cingul_Mid_Post	-3.02 (-7.43, 1.38)	0.178
lh_G_temp_sup_Lateral	-2.17 (-5.35, 1.01)	0.181
lh_G_temp_sup_G_T_transv	1.62 (-0.83, 4.08)	0.195
rh_G_oc_temp_med_Parahip	1.13 (-0.62, 2.87)	0.206
rh_S_pericallosal	-2.07 (-5.47, 1.33)	0.233
rh_G_front_sup	-2.73 (-7.25, 1.78)	0.236
lh_G_cingul_Post_ventral	0.90 (-0.73, 2.52)	0.281
rh_G_and_S_cingul_Ant	-2.05 (-5.89, 1.79)	0.294
rh_G_cingul_Post_dorsal	1.07 (-1.61, 3.74)	0.435
rh_Pole_temporal	-0.83 (-3.14, 1.48)	0.481
lh_S_temporal_inf	-1.03 (-4.38, 2.32)	0.547
rh_G_insular_short	0.76 (-1.93, 3.44)	0.581
lh_G_cingul_Post_dorsal	0.65 (-1.79, 3.09)	0.602
rh_G_temp_sup_Plan_tempo	-0.45 (-2.85, 1.96)	0.714
rh_S_oc_temp_med_and_Lingual	-0.51 (-4.01, 3.00)	0.777
lh_Pole_temporal	-0.35 (-2.99, 2.29)	0.793
rh_G_temp_sup_Plan_polar	-0.22 (-1.89, 1.45)	0.795
lh_G_temporal_middle	0.34 (-2.80, 3.49)	0.830
lh_S_pericallosal	0.20 (-2.60, 3.01)	0.887
rh_G_and_S_cingul_Mid_Ant	-0.30 (-4.64, 4.05)	0.893
lh_G_temp_sup_Plan_polar	-0.10 (-2.10, 1.91)	0.924

Table S6. Associations between hippocampus volumes and fatigue severity in patients with COVID-19.

Variable	Coefficient (95% CI)	Std. coefficient ß (95% CI)	p
Adjusted for sex and age			
Left Hippocampus	0.00 (-0.01, 0.01)	0.05 (-0.33, 0.42)	0.810
Right Hippocampus	0.00 (-0.01, 0.01)	0.09 (-0.27, 0.45)	0.615
Adjusted for sex, age and scanner type			
Left Hippocampus	0.00 (-0.01, 0.02)	0.04 (-0.34, 0.41)	0.842
Right Hippocampus	0.00 (-0.01, 0.01)	0.09 (-0.28, 0.46)	0.638
Adjusted for sex, age and study site			
Left Hippocampus	0.00 (-0.01, 0.02)	0.05 (-0.33, 0.43)	0.799
Right Hippocampus	0.00 (-0.01, 0.01)	0.10 (-0.28, 0.47)	0.617
Adjusted for sex, age and comorbidity			
Left Hippocampus	0.00 (-0.01, 0.01)	0.10 (-0.22, 0.42)	0.559
Right Hippocampus	0.00 (-0.00, 0.01)	0.16 (-0.15, 0.47)	0.314
Adjusted for sex, age and body mass index			
Left Hippocampus	0.01 (-0.01, 0.02)	0.15 (-0.23, 0.54)	0.434
Right Hippocampus	0.00 (-0.01, 0.02)	0.15 (-0.22, 0.52)	0.439

Table S7. Associations between hippocampus volumes and cognitive performance, in patients with COVID-19.

Variable	Coefficient (95% CI)	Std. coefficient ß (95% CI)	p
Adjusted for sex, age and years of education			
Left Hippocampus	0.000 (-0.003, 0.002)	-0.009 (-0.387, 0.369)	0.963
Right Hippocampus	0.000 (-0.002, 0.002)	0.061 (-0.306, 0.428)	0.746
Adjusted for sex, age, years of education and scanner type			
Left Hippocampus	-0.00 (-0.00, 0.00)	-0.01 (-0.40, 0.39)	0.963
Right Hippocampus	0.00 (-0.00, 0.00)	0.06 (-0.31, 0.43)	0.746
Adjusted for sex, age, years of education and study site			
Left Hippocampus	-0.00 (-0.00, 0.00)	-0.02 (-0.43, 0.38)	0.917
Right Hippocampus	0.00 (-0.00, 0.00)	0.05 (-0.33, 0.43)	0.789
Adjusted for sex, age, years of education and comorbidity			
Left Hippocampus	-0.00 (-0.00, 0.00)	-0.03 (-0.41, 0.34)	0.860
Right Hippocampus	0.00 (-0.00, 0.00)	0.02 (-0.35, 0.39)	0.911
Adjusted for sex, age, years of education and body mass index			
Left Hippocampus	-0.00 (-0.00, 0.00)	-0.04 (-0.49, 0.42)	0.870
Right Hippocampus	0.00 (-0.00, 0.00)	0.09 (-0.32, 0.50)	0.675

Table S8. Associations between hippocampus volumes and COVID-19 severity (WHO Ordinal Scale 4-6 [no invasive ventilation] vs. 7-9 [invasive ventilation]).

Region	Estimate	Std. Error	t-value	p
Adjusted for sex and age				
Left Hippocampus	-106.757	136.720	-0.781	0.438
Right Hippocampus	-219.670	165.624	-1.326	0.191
Adjusted for sex, age and scanner type				
Left Hippocampus	-152.778	143.365	-1.066	0.292
Right Hippocampus	-268.414	174.121	-1.542	0.129
Adjusted for sex, age and study site				
Left Hippocampus	-182.093	146.569	-1.242	0.220
Right Hippocampus	-308.178	177.595	-1.735	0.089
Adjusted for sex, age and comorbidity				
Left Hippocampus	-108.323	138.116	-0.784	0.436
Right Hippocampus	-221.319	167.340	-1.323	0.191
Adjusted for sex, age and body mass index				
Left Hippocampus	-100.020	167.859	-0.596	0.554
Right Hippocampus	-289.299	206.710	-1.400	0.168

Table S9. Associations between subcortical volumes and fatigue severity in patients after COVID-19, adjusted for sex and age.

Region	Coefficient (95% CI)	Std, coefficient ß (95% CI)	p
Left Caudate	-0.01 (-0.02, 0.01)	-0.18 (-0.59, 0.22)	0.382
Right Caudate	0.00 (-0.02, 0.01)	-0.13 (-0.52, 0.27)	0.533
Left Pallidum	0.00 (-0.03, 0.02)	-0.08 (-0.44, 0.28)	0.674
Right Pallidum	-0.02 (-0.04, 0.01)	-0.23 (-0.61, 0.15)	0.240
Left Putamen	-0.01 (-0.02, 0.01)	-0.23 (-0.73, 0.27)	0.373
Right Putamen	0.00 (-0.02, 0.01)	-0.20 (-0.67, 0.27)	0.413
Left Thalamus	0.00 (-0.01, 0.01)	-0.07 (-0.47, 0.33)	0.744
Right Thalamus	0.00 (-0.01, 0.01)	-0.11 (-0.53, 0.30)	0.590

Table S9a. Associations between subcortical volumes and fatigue severity in patients after COVID-19, adjusted for sex, age and scanner type.

Region	Coefficient (95% CI)	Std. coefficient ß (95% CI)	p
Left Caudate	-0.01 (-0.02, 0.01)	-0.18 (-0.55, 0.20)	0.368
Right Caudate	-0.00 (-0.02, 0.01)	-0.12 (-0.49, 0.25)	0.527
Left Pallidum	-0.01 (-0.03, 0.02)	-0.09 (-0.46, 0.28)	0.627
Right Pallidum	-0.02 (-0.04, 0.01)	-0.24 (-0.61, 0.13)	0.220
Left Putamen	-0.01 (-0.02, 0.01)	-0.21 (-0.65, 0.23)	0.359
Right Putamen	-0.00 (-0.02, 0.01)	-0.18 (-0.60, 0.24)	0.411
Left Thalamus	-0.00 (-0.01, 0.01)	-0.08 (-0.52, 0.36)	0.716
Right Thalamus	-0.00 (-0.01, 0.01)	-0.12 (-0.55, 0.31)	0.589

Table S9b. Associations between subcortical volumes and fatigue severity in patients after COVID-19, adjusted for sex, age and study site.

Region	Coefficient (95% CI)	Std. coefficient ß (95% CI)	p
Left Caudate	-0.01 (-0.02, 0.01)	-0.17 (-0.58, 0.24)	0.420
Right Caudate	-0.00 (-0.02, 0.01)	-0.11 (-0.52, 0.30)	0.605
Left Pallidum	-0.01 (-0.03, 0.02)	-0.09 (-0.46, 0.29)	0.655
Right Pallidum	-0.02 (-0.04, 0.01)	-0.24 (-0.62, 0.14)	0.218
Left Putamen	-0.01 (-0.02, 0.01)	-0.21 (-0.66, 0.24)	0.367
Right Putamen	-0.00 (-0.02, 0.01)	-0.17 (-0.61, 0.27)	0.452
Left Thalamus	-0.00 (-0.01, 0.01)	-0.08 (-0.53, 0.36)	0.721
Right Thalamus	-0.00 (-0.01, 0.01)	-0.12 (-0.55, 0.32)	0.602

Table S9c. Associations between subcortical volumes and fatigue severity in patients after COVID-19, adjusted for sex, age and comorbidity.

Region	Coefficient (95% CI)	Std. coefficient ß (95% CI)	p
Left Caudate	-0.00 (-0.01, 0.01)	-0.08 (-0.44, 0.28)	0.668
Right Caudate	-0.00 (-0.01, 0.01)	-0.02 (-0.37, 0.33)	0.908
Left Pallidum	0.00 (-0.02, 0.02)	0.02 (-0.29, 0.34)	0.883
Right Pallidum	-0.01 (-0.03, 0.01)	-0.20 (-0.53, 0.13)	0.251
Left Putamen	-0.00 (-0.02, 0.01)	-0.13 (-0.58, 0.31)	0.552
Right Putamen	-0.00 (-0.01, 0.01)	-0.03 (-0.45, 0.40)	0.904
Left Thalamus	-0.00 (-0.01, 0.00)	-0.15 (-0.50, 0.19)	0.397
Right Thalamus	-0.00 (-0.01, 0.00)	-0.07 (-0.43, 0.29)	0.691

Table S9d. Associations between subcortical volumes and fatigue severity in patients after COVID-19, adjusted for sex, age and body mass index.

Region	Coefficient (95% CI)	Std. coefficient ß (95% CI)	p
Left Caudate	-0.01 (-0.02, 0.01)	-0.23 (-0.63, 0.18)	0.280
Right Caudate	-0.00 (-0.02, 0.01)	-0.11 (-0.52, 0.30)	0.600
Left Pallidum	-0.00 (-0.02, 0.02)	-0.04 (-0.41, 0.33)	0.830
Right Pallidum	-0.01 (-0.04, 0.02)	-0.16 (-0.59, 0.28)	0.479
Left Putamen	-0.01 (-0.02, 0.00)	-0.34 (-0.83, 0.15)	0.187
Right Putamen	-0.01 (-0.02, 0.01)	-0.21 (-0.69, 0.28)	0.415
Left Thalamus	-0.00 (-0.01, 0.01)	-0.08 (-0.50, 0.34)	0.721
Right Thalamus	-0.00 (-0.01, 0.01)	-0.13 (-0.58, 0.31)	0.564

Table S10. Associations between subcortical volumes and cognitive performance in patients after COVID-19, adjusted for sex, age, and years of education.

Region	Coefficient (95% CI)	Std, coefficient ß (95% CI)	p
Left Caudate	0.000 (-0.003, 0.003)	0.03 (-0.41, 0.47)	0.903
Right Caudate	-0.001 (-0.004, 0.002)	-0.18 (-0.61, 0.24)	0.401
Left Pallidum	0.001 (-0.003, 0.005)	0.10 (-0.26, 0.47)	0.583
Right Pallidum	0.001 (-0.004, 0.005)	0.05 (-0.34, 0.43)	0.819
Left Putamen	0.000 (-0.003, 0.002)	-0.08 (-0.58, 0.43)	0.765
Right Putamen	0.000 (-0.002, 0.002)	0.06 (-0.39, 0.51)	0.781
Left Thalamus	0.000 (-0.001, 0.001)	0.05 (-0.35, 0.45)	0.799
Right Thalamus	0.000 (-0.001, 0.002)	0.07 (-0.35, 0.48)	0.755

Table S10a. Associations between subcortical volumes and cognitive performance in patients after COVID-19, adjusted for sex, age, years of education and scanner type.

Region	Coefficient (95% CI)	Std. coefficient ß (95% CI)	p
Left Caudate	0.00 (-0.00, 0.00)	0.03 (-0.41, 0.46)	0.903
Right Caudate	-0.00 (-0.00, 0.00)	-0.18 (-0.59, 0.23)	0.401
Left Pallidum	0.00 (-0.00, 0.01)	0.10 (-0.26, 0.47)	0.583
Right Pallidum	0.00 (-0.00, 0.01)	0.04 (-0.33, 0.42)	0.819
Left Putamen	-0.00 (-0.00, 0.00)	-0.08 (-0.58, 0.42)	0.765
Right Putamen	0.00 (-0.00, 0.00)	0.07 (-0.39, 0.53)	0.781
Left Thalamus	0.00 (-0.00, 0.00)	0.06 (-0.37, 0.48)	0.799
Right Thalamus	0.00 (-0.00, 0.00)	0.07 (-0.37, 0.51)	0.755

Table S10b. Associations between subcortical volumes and cognitive performance in patients after COVID-19, adjusted for sex, age, years of education and study site.

Region	Coefficient (95% CI)	Std. coefficient ß (95% CI)	p
Left Caudate	-0.00 (-0.00, 0.00)	-0.01 (-0.48, 0.47)	0.976
Right Caudate	-0.00 (-0.00, 0.00)	-0.26 (-0.72, 0.20)	0.273
Left Pallidum	0.00 (-0.00, 0.01)	0.10 (-0.27, 0.48)	0.587
Right Pallidum	0.00 (-0.00, 0.01)	0.05 (-0.34, 0.43)	0.814
Left Putamen	-0.00 (-0.00, 0.00)	-0.08 (-0.59, 0.43)	0.753
Right Putamen	0.00 (-0.00, 0.00)	0.05 (-0.44, 0.53)	0.856
Left Thalamus	0.00 (-0.00, 0.00)	0.06 (-0.37, 0.50)	0.778
Right Thalamus	0.00 (-0.00, 0.00)	0.08 (-0.37, 0.52)	0.734

Table S10c. Associations between subcortical volumes and cognitive performance in patients after COVID-19, adjusted for sex, age, years of education and comorbidity.

Region	Coefficient (95% CI)	Std. coefficient ß (95% CI)	p
Left Caudate	-0.00 (-0.00, 0.00)	-0.08 (-0.56, 0.39)	0.730
Right Caudate	-0.00 (-0.00, 0.00)	-0.30 (-0.74, 0.14)	0.193
Left Pallidum	0.00 (-0.00, 0.00)	0.07 (-0.30, 0.44)	0.712
Right Pallidum	0.00 (-0.00, 0.00)	0.01 (-0.38, 0.40)	0.961
Left Putamen	-0.00 (-0.00, 0.00)	-0.14 (-0.65, 0.37)	0.594
Right Putamen	-0.00 (-0.00, 0.00)	-0.01 (-0.48, 0.45)	0.952
Left Thalamus	0.00 (-0.00, 0.00)	0.06 (-0.33, 0.46)	0.754
Right Thalamus	0.00 (-0.00, 0.00)	0.05 (-0.36, 0.47)	0.802

Table S10d. Associations between subcortical volumes and cognitive performance in patients after COVID-19, adjusted for sex, age, years of education and body mass index.

Region	Coefficient (95% CI)	Std. coefficient ß (95% CI)	p
Left Caudate	0.00 (-0.00, 0.00)	0.01 (-0.49, 0.52)	0.955
Right Caudate	-0.00 (-0.00, 0.00)	-0.28 (-0.76, 0.20)	0.271
Left Pallidum	0.00 (-0.00, 0.01)	0.06 (-0.38, 0.50)	0.801
Right Pallidum	-0.00 (-0.01, 0.01)	-0.07 (-0.55, 0.42)	0.794
Left Putamen	-0.00 (-0.00, 0.00)	-0.22 (-0.80, 0.36)	0.469
Right Putamen	-0.00 (-0.00, 0.00)	-0.06 (-0.59, 0.48)	0.837
Left Thalamus	0.00 (-0.00, 0.00)	0.01 (-0.47, 0.49)	0.975
Right Thalamus	0.00 (-0.00, 0.00)	0.03 (-0.47, 0.52)	0.911

Table S11. Associations between subcortical volumes and COVID-19 severity (WHO Ordinal Scale 4-6 [no invasive ventilation] vs. 7-9 [invasive ventilation]), adjusted for sex and age.

Region	Coefficient (95% CI)	Z	p
Left Caudate	-0.001 (-0.003, 0.001)	-0.77	0.439
Right Caudate	-0.001 (-0.003, 0.001)	-0.75	0.451
Left Pallidum	-0.002 (-0.004, 0.001)	-1.17	0.244
Right Pallidum	-0.001 (-0.004, 0.003)	-0.36	0.722
Left Putamen	-0.001 (-0.003, 0.000)	-1.66	0.098
Right Putamen	-0.001 (-0.002, 0.001)	-1.21	0.225
Left Thalamus	-0.001 (-0.002, 0.000)	-2.39	0.017
Right Thalamus	-0.002 (-0.003, 0.000)	-2.57	0.010

Table S11a. Associations between subcortical volumes and COVID-19 severity (WHO Ordinal Scale 4-6 [no invasive ventilation] vs. 7-9 [invasive ventilation]), adjusted for sex, age and scanner type.

Region	Coefficient (95% CI)	Z	p
Left Caudate	-0.001 (-0.003, 0.001)	-0.92	0.359
Right Caudate	-0.001 (-0.003, 0.001)	-0.84	0.399
Left Pallidum	-0.003 (-0.006, 0.001)	-1.63	0.103
Right Pallidum	-0.001 (-0.004, 0.002)	-0.74	0.458
Left Putamen	-0.002 (-0.004, 0.000)	-1.86	0.063
Right Putamen	-0.001 (-0.002, 0.001)	-1.18	0.238
Left Thalamus	-0.002 (-0.003, -0.000)	-2.57	0.010
Right Thalamus	-0.002 (-0.004, -0.000)	-2.42	0.015

Table S11b. Associations between subcortical volumes and COVID-19 severity (WHO Ordinal Scale 4-6 [no invasive ventilation] vs. 7-9 [invasive ventilation]), adjusted for sex, age and study site.

Region	Coefficient (95% CI)	Z	p
Left Caudate	-0.001 (-0.003, 0.001)	-1.30	0.192
Right Caudate	-0.001 (-0.004, 0.001)	-1.36	0.172
Left Pallidum	-0.003 (-0.006, 0.001)	-1.59	0.111
Right Pallidum	-0.001 (-0.004, 0.002)	-0.56	0.575
Left Putamen	-0.002 (-0.004, 0.000)	-1.78	0.075
Right Putamen	-0.001 (-0.002, 0.000)	-1.41	0.159
Left Thalamus	-0.002 (-0.003, -0.000)	-2.58	0.010
Right Thalamus	-0.002 (-0.004, -0.000)	-2.45	0.014

Table S11c. Associations between subcortical volumes and COVID-19 severity (WHO Ordinal Scale 4-6 [no invasive ventilation] vs. 7-9 [invasive ventilation]), adjusted for sex, age and comorbidity.

Region	Coefficient (95% CI)	Z	p
Left Caudate	-0.001 (-0.003, 0.001)	-0.79	0.427
Right Caudate	-0.001 (-0.002, 0.001)	-0.77	0.444
Left Pallidum	-0.002 (-0.005, 0.001)	-1.19	0.233
Right Pallidum	-0.001 (-0.004, 0.003)	-0.33	0.741
Left Putamen	-0.001 (-0.003, 0.000)	-1.67	0.096
Right Putamen	-0.001 (-0.002, 0.000)	-1.29	0.198
Left Thalamus	-0.001 (-0.003, -0.000)	-2.37	0.018
Right Thalamus	-0.002 (-0.003, -0.000)	-2.59	0.010

Table S11d. Associations between subcortical volumes and COVID-19 severity (WHO Ordinal Scale 4-6 [no invasive ventilation] vs. 7-9 [invasive ventilation]), adjusted for sex, age and body mass index.

Region	Coefficient (95% CI)	Z	p
Left Caudate	-0.001 (-0.003, 0.001)	-0.86	0.388
Right Caudate	-0.001 (-0.003, 0.002)	-0.66	0.511
Left Pallidum	-0.001 (-0.005, 0.003)	-0.55	0.581
Right Pallidum	-0.001 (-0.006, 0.003)	-0.54	0.588
Left Putamen	-0.002 (-0.004, 0.001)	-1.51	0.131
Right Putamen	-0.000 (-0.002, 0.001)	-0.42	0.675
Left Thalamus	-0.001 (-0.002, 0.000)	-1.93	0.053
Right Thalamus	-0.002 (-0.004, -0.000)	-2.03	0.042

Table S12. Baseline characteristics of patients with missing and complete Fatigue Severity Scale (FSS).

Characteristic	FSS missing N = 15	FSS complete N = 42	p-value
Sex			0.354
male	8 (53%)	28 (68%)	
female	7 (47%)	13 (32%)	
Age (years)	53 ± 13	52 ± 13	0.909
Years of education	13.08 ± 3.73	15.28 ± 2.36	0.064
Comorbidity			0.523
none	6 (40%)	13 (31%)	
any	9 (60%)	29 (69%)	

Table S13. Baseline characteristics of patients with missing and complete Montreal Cognitive Assessment (MoCA).

Characteristic	MoCA missing N = 17	MoCA complete N = 40	p-value
Sex			>0.999
male	11 (65%)	25 (64%)	
female	6 (35%)	14 (36%)	
Age (years)	50 ± 10	54 ± 14	0.276
Years of education	14.86 ± 2.68	14.69 ± 2.98	0.850
Comorbidity			0.152
none	8 (47%)	11 (28%)	
any	9 (53%)	29 (73%)	
